# Comparison of Serological and Molecular Methods With High-Throughput Sequencing for the Detection and Quantification of Grapevine Fanleaf Virus in Vineyard Samples

**DOI:** 10.3389/fmicb.2018.02726

**Published:** 2018-11-22

**Authors:** Emmanuelle Vigne, Shahinez Garcia, Véronique Komar, Olivier Lemaire, Jean-Michel Hily

**Affiliations:** L’UMR Santé de la Vigne et Qualité du Vin, INRA-Université de Strasbourg, Colmar, France

**Keywords:** grapevine, GFLV, detection, serological and molecular methods, high-throughput sequencing, contamination evaluation protocol

## Abstract

Grapevine fanleaf virus (GFLV) is the main causal agent of fanleaf degeneration, the most damaging viral disease of grapevine. GFLV is included in most grapevine certification programs that rely on robust diagnostic tools such as biological indexing, serological methods, and molecular techniques, for the identification of clean stocks. The emergence of high throughput sequencing (HTS) offers new opportunities for detecting GFLV and other viruses in grapevine accessions of interest. Here, two HTS-based methods, *i.e.*, RNAseq and smallRNAseq (focusing on the 21 to 27 nt) were explored for their potential to characterize the virome of grapevine samples from two 30-year-old GFLV-infected vineyards in the Champagne region of France. smallrnaseq was optimal for the detection of a wide range of viral species within a sample and RNAseq was the method of choice for full-length viral genome assembly. The implementation of a protocol to discriminate between low GFLV titer and *in silico* contamination (intra-lane contamination due to index misassignment) during data processing was critical for data analyses. Furthermore, we compared the performance of semi-quantitative DAS-ELISA (double antibody enzyme-linked immunosorbent assay), RT-qPCR (Reverse transcription-quantitative polymerase chain reaction), Immuno capture (IC)-RT-PCR, northern blot for viral small interfering RNA (vsiRNA) detection and RNAseq for the detection and quantification of GFLV. While detection limits were variable among methods, as expected, GFLV diagnosis was consistently achieved with all of these diagnostic methods. Together, this work highlights the robustness of DAS-ELISA, the current method routinely used in the French grapevine certification program, for the detection of GFLV and offers perspectives on the potential of HTS as an approach of high interest for certification.

## Introduction

More than 80 viruses and five viroids have been identified in *Vitis spp*., making grapevine the most virus-infected agricultural commodity known to date (Martelli, 2017). While some viruses might not be directly linked to a particular disease, others are of great economic concern to the grapevine industry. The grapevine fanleaf virus (GFLV) from the genus *Nepovirus* in the family *Secoviridae* (Sanfaçon, 2015) causes fanleaf degeneration disease (Schmitt-Keichinger et al., 2017). This disease is of major economic concern (Andret-Link et al., 2004). The genome of GFLV is composed of two single-stranded positive-sense RNA molecules (RNA1 and RNA2). Both genomic RNAs are necessary for infection and can be associated with a satellite RNA (RNA3) (Schmitt-Keichinger et al., 2017). GFLV exhibits strong genetic diversity with genome sequences being divergent up to 20%, with many recombination events being identified on both genomic RNAs from field isolates (Vigne et al., 2004, 2008; Zhou et al., 2017; Hily et al., 2018c).

Preventive measures based on the use of clean grapevines derived from certified as free of GFLV stocks is the most reliable option to manage fanleaf disease. The identification of clean vines through extensive and robust diagnosis is foundational to certification schemes (Golino et al., 2017a,b). GFLV and other viruses need to be undetectable in order for vines to be certified. Several diagnostic methodologies are available for GFLV such as bioassays, serological assays and polymerase chain reaction (PCR)-based assays (Golino et al., 2017a). A combination of both ELISA and PCR-based techniques, known as immunocapture-PCR (IC-PCR), is also used for GFLV detection. Recently, high-throughput sequencing (HTS) is explored as a new approach for virus discovery and detection. HTS has the potential for inclusion in certification programs (Al Rwahnih et al., 2015; Maree et al., 2018). Different nucleic acid preparations and different sequencing platforms are routinely used for HTS (Adams et al., 2009; Al Rwahnih et al., 2009; Kreuze et al., 2009). The benefits and pitfalls of each of them have been extensively reviewed (Kircher and Kelso, 2010; Barzon et al., 2013; Reuter et al., 2015; Roossinck et al., 2015). Briefly, a virus and its genetic make-up can be detected by (i) characterizing total RNA or DNA, with or without specific enrichment steps (Dayaram et al., 2012; Beuve et al., 2018), (ii) double-stranded (ds) RNA for viruses with dsRNA genomes and RNA viruses and viroids that form dsRNA during their replication (Coetzee et al., 2010; Blouin et al., 2016), (iii) products of RNA interference, an adaptive antiviral plant defense mechanism, such as vsiRNA (viral small-interfering RNA) (Donaire et al., 2009; Kreuze et al., 2009), and (iv) encapsidated nucleic acids using the VANA (Virion-Associated Nucleic Acid) approach (Filloux et al., 2015). HTS revolutionizes grapevine virus diagnostics with unbiased and massive sequencing, compared to serological and molecular assays which rely on specific antibodies or primers/probes (Al Rwahnih et al., 2015). However, prior to the adoption of HTS for routine and generic viral detection, many challenges have to be overcome and some factors need to be optimized such as: (i) the development of nucleic acid preparation protocols for consistent diagnostic results, (ii) the development of straightforward and easy-to-use bioinformatics tools, and (iii) the identification of contaminations such as ‘between-run’ contaminations or ‘intra-lane’ cross contamination, also known as ‘index hopping.’ The former type of contaminations derives from a carry-over of contaminants from previous runs, while the latter arises when multiple libraries are pooled in a single lane and sequenced simultaneously (Kircher et al., 2012; Maree et al., 2018).

**FIGURE 1:**
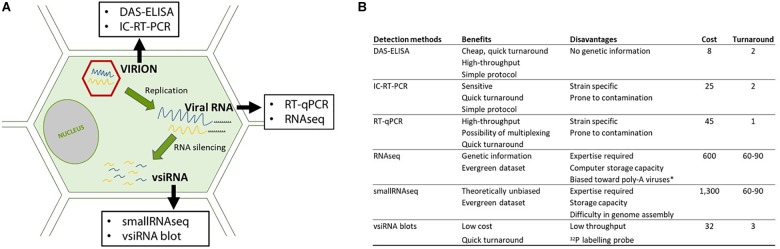
Details on the detection methods used in this study. **(A)** Detection methods directed at different targets (virions: in red and RNAs: in blue and yellow) along the viral cycle and **(B)** benefits and pitfalls of each methodology. Cost (in euro) per sample taking into account all steps required for the diagnosis, from sample management, buffers, enzymes as well as the time of the manipulator such as a specialized person for performing and analyzing the HTS data. Turnaround is expressed in days from sample reception to result delivery. ^∗^In our study, a poly-A selection was performed prior to sequencing.

In this study, we compared HTS, including RNAseq and smallRNAseq, with serological and molecular methods for the detection of GFLV. These methods were selected to maximize GFLV detection by targeting viral particles (DAS-ELISA); encapsidated viral RNAs (IC-RT-PCR), viral RNAs (RT-qPCR and RNAseq) and vsiRNAs (siRNA blots and small RNAseq (Figure 1A). We also developed a bioinformatics framework to identify intra-lane cross contamination for GFLV.

## Materials and Methods

### Plant and Viral Material

Samples of young apical leaves were collected from 20 selected vines in two 30-year-old vineyards of *Vitis vinifera* cv. Chardonnay highly affected by fanleaf degeneration in the Champagne region (France) (Supplementary Table S1). Samples were collected in June when GFLV titer is high (Walter et al., 1984; Walter and Etienne, 1987) and flash frozen. Tissues were ground and homogenized into a fine powder with a mortar and pestle in liquid nitrogen. Aliquots of ground tissue were stored at −80°C and used for further testing by DAS-ELISA, IC-RT-PCR, RT-qPCR, vsiRNA Northern blot, and RNAseq.

Another set of six GFLV-infected grapevine leaf samples was chosen to compare the performance of RNAseq and smallRNAseq. Four samples (IC-MaA8191 and IC-MaA8193 from *V. vinifera* cv. Grenache, and IC-P1a and IC-P2a from *V. vinifera* cv. Chardonnay) were from the INRA grapevine virus collection in Colmar, France; and two samples (Va1 and Va4 from *V. vinifera* cv. Chardonnay) were from a commercial vineyard in Bagneux-la-Fosse in the Champagne region of France. Two samples (ENTAV-E39 and ENTAV-E173 from *V. vinifera* cv. 110R and Grenache, respectively) were from the grapevine collection in the Institut Français de la Vigne et du Vin (ENTAV-IFV, Le Grau-du-Roi, France). Additionally, leaf samples of grapevines infected by GFLV isolates GHu, F13-Col, B844, and CO1 (A17b) and IC-MaA8193 (Supplementary Table S2) from the INRA grapevine virus collection in Colmar, France were selected to verify the robustness of the diagnosis methods tested in this study. Leaves collected from these GFLV-infected grapevines were prepared in ELISA grinding buffer and conserved at-20°C until further testing. Sequences of these GFLV isolates and other GFLV isolates that were used as reference for bioinformatics purposes in this study are listed in Supplementary Table S2.

### Total RNA Extraction From Grapevine Leaf Samples and High Throughput Sequencing

Total RNAs were extracted from 100 mg of grapevine leaf tissue using an adapted protocol for the recovery of small and high molecular weight RNAs with the RNeasy Plant mini kit (Qiagen, Venlo, Netherlands), as per the manufacturer’s recommendations. Post-extraction, purity criteria (A260_nm_/A230_nm_ and A260_nm_/A280_nm_ both > 1.8) and quality levels (RNA integrity number > 8) were assessed via a Bioanalyzer (Agilent, Santa Clara, CA, United States). Total RNA was used to prepare cDNA libraries after a poly-A selection at the GeT-PlaGe Genotoul platform facility (INRA-Toulouse, France). The HTS approach of choice in this study was a paired-end 2 × 150 pb RNAseq completed on a Hiseq 3000 (Illumina, San Diego, CA, United States) following the manufacturer’s instructions. Double indexing was used for some samples (Lane 1, Supplementary Table S1) while single indexing was performed for other samples (Lane 2, Supplementary Table S1). For siRNA sequencing, samples were sequenced on a Hiseq Instrument at 1 × 50 pb and multiplexed at Fasteris (Plan-les-Ouates, Switzerland).

### HTS Data Analyses

Demultiplexing was performed by GeT-Genotoul and Fasteris. The GeT-Genotoul platform used Bcl2fastq version 2.20.0.422, allowing 0 barcode mismatches with a minimum trimmed read length of 35. Analyses of datasets were performed using Workbench 11.0 software (CLC Genomics Workbench, Aarhus, Denmark). Prior to any downstream analyses, a FastQC was performed. For RNAseq datasets, only reads above 70 nucleotides (nts) were kept after trimming and quality check (Supplementary Tables S3, S4). For smallRNAseq datasets, only reads between 20 and 27 nt were kept for downstream analyses after a quality check step (Supplementary Table S4).

The sanitary status of grapevine samples was assessed by mapping reads to a collection of curated reference sequences of viruses known to infect grapevine (Martelli, 2017), as previously described (Hily et al., 2018c). Briefly, relaxed mapping stringency (0.5/0.7 or 0.9/0.9, corresponding to read length/similarity parameters for RNAseq and smallRNAseq data, respectively) was used to span maximum genome diversity within virus species. RPKM (Read Per Kilobase Million) was used as normalization method. RPKM values express abundance of viral RNAs, and are obtained by accounting for the number of reads mapping a virus genome, the genome size of the virus and the total number of reads from the sample. *De novo* assemblies (parameters of word size of 17 and minimum contig length of 200) were performed after removal of reads that mapped to the *Vitis vinifera* genome^1^. Contigs were then tested against GenBank reference sequences using Blastn/Blastx^2^.

For the detection of possible intra-lane contamination, all reads that mapped to the GFLV consensus RNA1 and RNA2 sequences at relaxed stringency parameters (see above) were recovered from each sample. Reads were then mapped to GFLV sequences obtained from the same sequencing lane (e.g., IC-MaA8193, IC-P1a, IC-P2a, KX034936, and KX034888) or GFLV sequences that were not present in the same lane [F13-col, B844, CO1 (A17b), CO2 (A17d), and GHu] (Supplementary Tables S1, S2). Mapping was performed at a very high stringency in order to confirm the origin of the reads [0.95/0.98 and 0.99/0.99 of (length/similarity) for RNAseq and smallRNAseq datasets, respectively]. A schematic representation of our protocol for detecting possible contamination is shown as Supplementary Data Sheet 1.

### RT-qPCR for GFLV Diagnosis

For viral detection and absolute quantification with RT real-time PCR, 20 ng of total RNA, quantified by Nanodrop^TM^, were reverse-transcribed into cDNA using Superscript III reverse transcriptase (Invitrogen, Carlsbad, CA, United States) with a mix of oligo (dT). Eva Green qPCR mix (Biorad^TM^) was used with specific primers targeting GFLV-RNA1 and RNA2 for real time amplification (Biorad^TM^ CFX96 real-time system). Degenerated primers (Supplementary Table S5) were designed within the most conserved regions after alignment of 32 complete GFLV genomes (from NCBI and our own sequence database) for the amplification of about 120 bp fragments. PCR was performed in 96-well optical reaction plates for 30 s at 95°C, 40 cycles of denaturation for 5 s at 95°C, annealing and elongation for 5 s at 65°C. Each sample/primers combination was carried out in triplicate. A melting curve analysis was performed to ascertain that a single product was produced in each well. Viral quantification was expressed as molecule of viral RNA per ng of total RNA. The detection threshold of GFLV was determined via a serial dilution of plasmids containing cDNA from GFLV RNA1 and RNA2 (50 to 5.10^−7^ ng of DNA) (Vigne et al., 2013).

### Semi-Quantitative DAS-ELISA for GFLV Diagnosis

DAS-ELISA was performed using a commercial kit (Bioreba AG, Reinach, Switzerland), as previously described (Vigne et al., 2013). For each sample, 100 μL of a mixture of frozen tissue powder (0.5 g) re-suspended in 5 mL of extraction buffer were loaded into microtiter plates, as well as serial dilution 10^−1^ and 10^−2^ for each sample. For quantification purpose, each microplate contained a serial dilution of a purified GFLV preparation, ranging from 7,480 to 0.22 pg of virion per well. GFLV concentration for each sample was deduced from a linear regression.

### IC-RT-PCR for GFLV Diagnosis

For each DAS-ELISA performed, a second plate was run in parallel for IC-RT-PCR. After antibody/antigen incubation, 12 μl of sterile water were added for 10 min at 70°C and 5 min on ice to disrupt viral particles. The recovered viral RNAs were subjected to first-strand cDNA synthesis with degenerated primers (Supplementary Table S5) to amplify fragments of 387 pb for RNA1 and 567 pb for RNA2. The cycling PCR parameters were an initial denaturation at 95°C for 2 min, followed by 36 cycles of 30 s at 95°C, 30 s at 52°C and 45 s at 72°C, followed by a 5 min at 72°C. For IC-RT-qPCR, after recovering the encapsidated viral RNAs, the aforementioned RT-qPCR protocol was applied.

### Northern Blot for GFLV vsiRNAs Detection

Total RNA was extracted from 0.1 g of frozen tissue using 1 ml Concert Plant RNA Reagent following manufacturer’s protocol (Invitrogen, Carlsbad, CA, United States). Low molecular weight RNA was analyzed using 30 μg of total RNA separated by denaturing polyacrylamide gel electrophoresis and transferred to Hybond N + membranes (GE Healthcare, Piscataway, NJ, United States). Following chemically crosslinking with EDC treatment (Pall et al., 2007), hybridization was done with a mix of five radiolabeled DNA probes amplified from five genetically distinct GFLV isolates (F13-col, B844, CO1 (A17b), GHu, and IC-MaA8193) in siRNA hot spot regions of RNA1 and RNA2 according to our smallRNAseq datasets (Supplementary Table S5). DNA probes labeled with α-32P dCTP (Hartmann, Braunschweig, Germany) were obtained using the Prime-a-Gene^®^ labeling system (Promega, Madison, WI, United States). Hybridization was conducted overnight at 42°C in PerfectHyb^TM^ plus buffer (Sigma-Aldrich, St Louis, MO, United States). Washes were done three times for 10 min at 50°C in 2X SSC and 2% SDS and the membranes were exposed to Biorad FX phosphorimager (Biorad, Hercules, CA, United States). To determine the detection threshold of vsiRNA blot, we used 21 and 24 nt RNA oligonucleotides specific to RNA1 or RNA2 (Supplementary Table S5). We performed a serial dilution to test the detection level for each RNA oligonucleotide. The loading control was hybridized with a vvi-miR159c probe (Supplementary Table S5).

## Results

### Comparison of RNAseq and smallRNAseq

We tested and compared two HTS techniques to detect viruses in grapevine samples (Figure 1) with RNAseq (Illumina 3000, 2 × 150 bp) detecting mostly poly-A-tailed virus sequences and smallRNAseq (1 × 50 bp) detecting vsiRNA as the final product of the adaptive antiviral plant defense mechanism (Hamilton et al., 2002). Samples from four grapevines from the INRA grapevine collection in Colmar, France (three being GFLV-infected and one appearing as non-infected by GFLV) and from two grapevines showing typical fanleaf symptoms (stunting and very short internodes) in a Champagne vineyard were selected for this study. Two grapevine accessions provided by ENTAV-IFV were considered as negative controls. Our main goal was to detect GFLV and other viruses. It is important to mention that the eight samples were multiplexed in the same sequencing lane in the two HTS techniques tested (Supplementary Table S1).

### GFLV Detection

The same strategy was used to analyze the RNAseq and smallRNAseq datasets with most detection parameters being relaxed for the recovery of maximal GFLV diversity. Although GFLV is genetically diverse (Vigne et al., 2004; Mekuria et al., 2009; Zhou et al., 2017), we mapped all reads to RNA1 and RNA2 consensus sequences obtained from seven GFLV genomes (Hily et al., 2018c). As a result, many reads mapping to GFLV consensus sequences were recovered in all samples tested (Table 1) with 75 reads mapping to the GFLV-RNA1 consensus sequence for sample ENTAV-E39 and 183,385 reads mapping to the GFLV-RNA2 consensus sequence for sample IC-P1a for the RNAseq dataset; and with 254 (sample IC-MaA8191) to 797,497 (sample IC-MaA8193) reads for the smallRNAseq dataset (Table 1). The eight samples used for this study had either a ‘high read count’ (HRC) with elevated reads (67,540 to 797,497) mapping to the GFLV consensus sequences (highlighted in gray in Table 1) or a ‘low read count’ (LRC) with less than 1,000 reads recovered from either HTS techniques. Such distinct contrast in read recovery, ranging from 1 to 10,000 fold, was specific to GFLV; it was not observed when mapping other virus genomes. For example, 0 to 6 reads mapped to the genome of grapevine virus A (GVA; whose genome size is close to that of GFLV RNA1) and 671 to 11,620 reads mapped to the genome of grapevine rupestris stem pitting-associated virus (GRSPaV), an ubiquitous virus of grapevine (Table 1).

**Table 1:** Information about reads mapping to viral sequences.

				GFLV consensus						GFLV *de novo* sequence				GVA	GRSPaV
				#reads		Highest depth		Coverage %		reads		Coverage %		# reads	# reads
Sample name	DAS-ELISA	Technique	Total read #	RNA1	RNA2	RNA1	RNA2	RNA1	RNA2	RNA1	RNA2	RNA1	RNA2		
IC-MaA8191		small RNAseq	18,343,185	**374**	**254**	**29**	**27**	**38.2**	**38.7**	–	–	–	–	2	6,631
	–	RNAseq	165,823,267	**482**	**783**	**26**	**42**	**98.1**	**96.4**	–	–	–	–	0	33,799
IC-MaA8193	+	small RNAseq	20,366,738	*797,497*	*518,542*	*46,686*	*22,620*	*56.5*	*48.4*	*2,259,514*	*1,593,089*	*99.9*	*99.8*	0	6,528
		RNAseq	23,461,101	*93,017*	*167,810*	*5,009*	*5,195*	*100.0*	*97.9*	*96,756*	*182,407*	*100.0*	*100.0*	4	4,092
ENTAV-E39	–	small RNAseq	18,161,215	**835**	**500**	**68**	**43**	**41.4**	**45.8**	–	–	–	–	0	671
		RNAseq	22,164,625	**75**	**97**	**8**	**21**	**58.7**	**82.1**	–	–	–	–	0	14
ENTAV-E173	–	small RNAseq	23,226,315	**468**	**329**	**37**	26	41.6	38.1	–	–	–	–	1	3,038
		RNAseq	51,911,501	**243**	**265**	**20**	24	87.2	93.5	–	–	–	–	0	3,030
IC-P1a	+	small RNAseq	27,468,283	*711,941*	*396,013*	*54,488*	*68,134*	*81.5*	*71.7*	*1,879,692*	*1,233,456*	*99.7*	*99.6*	1	3,561
		RNAseq	29,519,363	*155,869*	*183,385*	*8,374*	*14,409*	*99.8*	*97.6*	*165,675*	*204,211*	*100.0*	*100.0*	0	3,176
IC-P2a	+	small RNAseq	22,989,799	*591,793*	*381,518*	*45,195*	*64,694*	*82.1*	*73.3*	*1,844,236*	*1,097,753*	*99.5*	*99.8*	6	11,097
		RNAseq	22,461,740	*67,540*	*101,207*	*4,724*	*7,753*	99.8	97.6	*68,942*	*112,932*	*100.0*	*100.0*	0	5,498
Va1	–	small RNAseq	24,068,532	388	255	27	34	36.4	34.8	–	–	–	–	0	5,971
		RNAseq	11,633,150	130	191	10	58	78.8	94.7	–	–	–	–	0	3,833
Va4	–	small RNAseq	29,827,959	545	345	37	38	42.5	42.4	–	–	–	–	4	11,620
		RNAseq	8,596,909	127	187	12	70	81.5	94.1	–	–	–	–	0	10,805

*The number of reads (# reads) mapping to the consensus grapevine fanleaf virus (GFLV) RNA1 and RNA2 sequences obtained from seven full-length GFLV genomes (Hily et al., 2018c) are indicated. The highest depth corresponds to the highest number of reads present at any position along the genome. Coverage % corresponds to the coverage of the reference. In bold are numbers of reads from ‘low read count’ samples and numbers of reads from ‘high read count’ samples are in italic and highlighted in gray. For GFLV positive samples, de novo assembled GFLV sequences were used as reference (– : not applicable since no genome could be assembled). Sequences of grapevine virus A (GVA) and grapevine rupestris stem pitting-associated virus (GRSPaV) were used as controls.*

Sequencing depth was proportional to the number of reads mapped to each GFLV sequence. By focusing on the genome coverage percentage, close to the full-length GFLV genome sequence was covered by reads from the RNAseq dataset for HRC samples (97.6 to 100% genome coverage) and, to a lesser extent, for LRC samples (58.7–98.1% genome coverage). The difference in genome coverage between the two categories of samples was also observed with the smallRNAseq dataset but with a lower coverage (48.4–82.1 and 34.8–45.8% for HRC and LRC samples, respectively). While the number of reads was antithetical between HRC and LRC samples, their presence was undeniable, covering a high percentage of the GFLV genome. These results begged the following questions: Is the GFLV titer in LRC samples extremely low? Or, do these reads correspond to some contamination due to multiplexing? These two questions are of particular interest for the two ENTAV-IFV samples: ENTAV-E39 and ENTAV-E173 which are resulting from a successful therapeutics treatment via microshoot tip culture (Golino et al., 2017b), never displayed any disease symptom and always tested negative for major viral diseases of grapevine.

### A Protocol to Identify GFLV Intra-Lane Contamination

**FIGURE 2:**
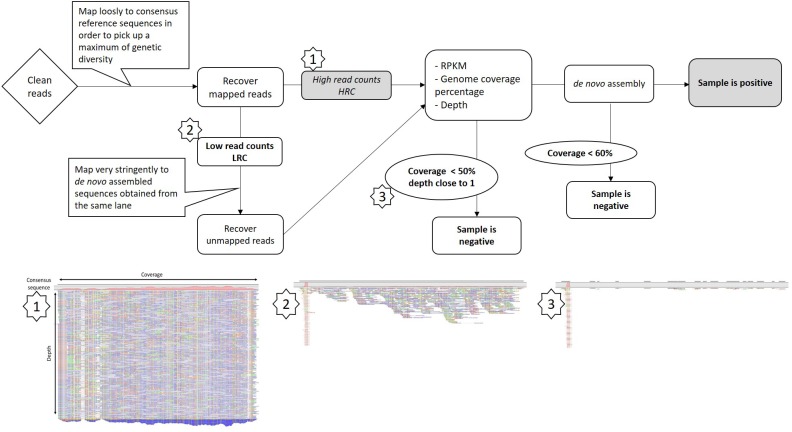
Framework for Grapevine fanleaf virus (GFLV) intra-lane contamination detection from RNAseq libraries. Images below are taken from the CLC Workbench software and correspond to steps within the framework specifically looking at GFLV RNA2. A more detailed protocol is provided in Supplementary Data Sheet 1.

To discriminate between low GFLV titer or intra-lane contamination due to the sample multiplexing step, we designed a framework of basic bioinformatics steps for RNAseq datasets (Figure 2). First, all reads mapping to GFLV consensus sequences at low stringency (parameters 0.5/0.7, length/identity, see section “Materials and Methods”) were recovered. Reads were then mapped to a series of beforehand *de novo* assembled GFLV genomes that were either present within the same sequencing lane (e.g., IC-P1a, IC-P2a and IMA-GMO) (Supplementary Tables S1, S2) or to GFLV sequences that were not present on the same sequencing lane (e.g., F13-col and B844) (Supplementary Table S2). For this step, very stringent parameters (0.95/0.98) were used (Table 2). Even at extremely high stringency and using only a small portion of GFLV sequences recovered from the same sequencing lane, 69–80% reads for GFLV RNA1 and 60–96% reads for GFLV RNA2 corresponded to these sequences (Table 2). In contrast, from the same pool of reads, only 0–2% of them mapped to sequences from GFLV variants not present in the sequencing lane, with the exception of 24% for GFLV RNA1 of sample IC-MaA819. This dichotomy in percentages indicates a probable source of contamination from samples being multiplexed within a particular sequencing lane. This was true also for smallRNAseq datasets although a higher percentage of reads mapping to GFLV variant sequences from either the same (88–99%) or different (26–52%) sequencing lanes was obtained. The higher percentage obtained with smallRNAseq compared to RNAseq datasets could be explained by the fact that smaller sequences (20–27 nt vs. ≈ 146 nt, Supplementary Table S4) allowed for more reads to be mapped onto a genome by the former compared to the latter technology.

**Table 2:** Assessment of GFLV intra-lane contamination in RNAseq and smallRNAseq datasets.

			RNA1			RNA2		
			GFLV	Same lane	Other lane	GFLV	Same lane	Other lane
Sample name	read #	Technique	Consensus	Variants	Variants	Consensus	Variants	Variants
IC-MaA8191	18,343,185	smallRNAseq	374	340 (91%)	121 (32%)	254	223 (88%)	67 (26%)
	165,823,267	RNAseq	482	395 (80%)	115 (24%)	783	472 (60%)	14 (2%)
ENTAV-E39	18,161,215	smallRNAseq	835	816 (98%)	382 (46%)	500	480 (96%)	186 (37%
	22,164,625	RNAseq	75	60 (80%)	1 (1%)	97	63 (65%)	0 (0%)
ENTAV-E173	23,226,315	smallRNAseq	468	461 (99%)	220 (47%)	329	318 (97%)	121 (37%)
	51,911,501	RNAseq	243	178 (73%)	5 (2%)	265	223 (84%)	2 (1%)
Va1	24,068,532	smallRNAseq	388	377 (97%)	203 (52%)	255	247 (97%)	106 (42%)
	11,633,150	RNAseq	130	99 (76%)	2 (2%)	191	126 (66%)	0 (0%)
Va4	29,827,959	smallRNAseq	545	524 (96%)	270 (50%)	345	330 (96%)	141 (41%)
	8,596,909	RNAseq	127	87 (69%)	3 (2%)	187	180 (96%)	0 (0%)

*Reads that first mapped to GFLV-consensus sequences are indicated (Table 1). They were recovered and then mapped to *de novo* assembled sequences (RNA1 and RNA2) obtained from positive samples present on the same sequencing lane (same lane variants) or to sequences absent from that lane (other lane variants, Supplementary Tables S1, S2). Number of reads and percentage (in parenthesis) that mapped are indicated. Mapping parameters were set very stringently, 0.95/0.98 and 0.99/0.99 (length/identity) for RNAseq and smallRNAseq datasets, respectively.*

Our ‘intra-lane contamination’ protocol was applied to all LRC samples. Consequently, the intra-lane GFLV contaminant reads were removed from the datasets and all LRC samples were ultimately defined as negative (Figure 2 and Table 1). Even if a contamination might be observed for other viruses (see GRSPaV in sample ENTAV-E39, Table 1), this protocol could not be used in our datasets for viruses other than GFLV since their accumulation was not sufficient enough to trigger the implementation of the ‘intra-lane contamination’ method. For example, the number of reads mapping to GRSPaV sequences corresponded to only 1.1–4% of those mapping GFLV sequences in samples IC-MaA8193, IC-P1a and IC-P2a (Table 1).

### Analysis of the Sanitary Status of Grapevine Samples: Detection of Viruses and Identification of Viral Variants

The robustness of RNAseq and smallRNAseq for the detection of viruses other than GFLV and viroids was tested. Two methods were applied (Hily et al., 2018c): a direct mapping using a set of references and a Blastn/Blastx analysis following a *de novo* assembly. For most samples, the same viral species were detected by both HTS techniques (Table 3). GRSPaV, as well as hop stunt viroid (HSVd) and grapevine yellow speckle viroid-1 (GYSVd1), were readily detected and assembled from all samples, except for sample ENTAV-E39 for which only HSVd was detected. Also, it was not surprising to see that a grapevine leafroll-associated virus 3 (GLRaV-3), which does not have a poly-A-tailed genome, was detected by smallRNAseq but not by RNAseq in sample IC-MaA8193 (Table 3 and Supplementary Figure S3). Nonetheless, some viruses with a non-poly-A-tailed genome were detected via RNAseq probably because the viral titer was high or the viral sequence might contain a stretch of adenine that was selected by the poly-(A) selection process (see GLRaV2 and GLRaV3 in samples Va4 and IC-MaA8191, respectively, Table 3). What was unexpected was that grapevine Red Globe virus (GRGV), a virus with a poly-A-tailed genome from the family *Tymoviridae*, was not detected by RNAseq in sample Va4 but identified using smallRNAseq (Table 3 and Supplementary Figure S3).

**Table 3:** Virus detection in eigth grapevine samples by RNAseq and smallRNAseq.

		GFLV						
Sample name	Technique	RNA1	RNA2	GRSPaV	GYSVd1	HSVd	GFkV	GRVFV	GRGV	GLRaV2	GLRaV3
IC-MaA8191	smallRNAseq	–	–	✓	✓	✓	–	–	–	–	1
	RNAseq	–	–	2	1	1	–	–	–	–	✓
IC-MaA8193	smallRNAseq	✓	✓	✓	✓	✓	–	–	–	–	1^∗∗^
	RNAseq	1	1	1	1	1	–	–	–	–	**–^∗∗^**
ENTAV-E39	smallRNAseq	–	–	–	–	✓	–	–	–	–	–
	RNAseq	–	–	–	–	1	–	–	–	–	–
ENTAV-E173	smallRNAseq	–	–	✓	✓	✓	–	–	–	–	–
	RNAseq	–	–	1	1	1	–	–	–	–	–
IC-Pa1	smallRNAseq	✓	✓	✓	✓	✓	–	✓	✓	–	–
	RNAseq	1	1	2	2	1	–	2	✓	–	–
IC-P2a	smallRNAseq	✓	✓	✓	✓	✓	–	–	✓	–	–
	RNAseq	1	1	4	1	1	–	–	✓	–	–
Va1	smallRNAseq	–	–	✓	✓	✓	1	–	–	–	–
	RNAseq	–	–	2	2	1	1	–	–	–	–
Va4	smallRNAseq	–	–	✓	✓	✓	1	–	1^∗^	1	–
	RNAseq	–	–	3	1	1	1	–	–^∗^	✓	–

*Names of viruses detected (√) and number of genomes assembled (# and highlighted in gray). ^∗^Sample Va4 showed 281,643 reads for grapevine Red Globe virus (GRGV) in the smallRNAseq datasets and 118 reads (23.6% genome covered) in RNAseq datasets. See Supplementary Figure S3 for more information. ^∗∗^Sample IC-MaA8193 showed 60,875 reads for grapevine leafroll-associated virus 3 (GLRaV3) in the smallRNAseq datasets and 10 reads (4.71% genome covered) in the RNAseq datasets. See Supplementary Figure S3 for more information.*

From this study, it seems that smallRNAseq libraries allow for the detection of more viral species than the RNAseq technique. However, complete viral genome sequences were rarely obtained via *de novo* assembly when using the smallRNAseq datasets because many genome regions were not covered by reads. In contrast, it was very straightforward to obtain complete (to near complete) viral genomes using RNAseq datasets. Furthermore, we were able to assemble and identify different variants of a virus species (GRSPaV and GRVFV) within the same sample from either libraries (Table 3), however, *de novo* assembly was much easier from RNAseq compared to smallRNAseq datasets. No new viral species were detected in any of the samples selected for this study using the RNAseq or smallRNAseq datasets.

### RNAseq Versus Traditional GFLV Detection Methods

We compared the performance of RNAseq, DAS-ELISA, RT-qPCR, IC-RT-PCR and vsiRNA blot for GFLV diagnosis using grapevine leaf samples from the INRA grapevine virus collection in Colmar (France) and from two vineyards in the Champagne region of France. Five GFLV isolates from the INRA grapevine virus collection were selected to identify the most robust broad-spectrum diagnostic methodology. These five GFLV isolates have 82–92% nucleotide sequence identity among their RNA1 and RNA2 (Supplementary Figure S1).

### Traditional Methods for GFLV Diagnosis

First, we compared the sensitivity of each technics by testing serial dilutions of GFLV-infected grapevine leaf crude sap from five GFLV isolates. All isolates were readily detected by three methods (Table 4). However, IC-RT-qPCR that was designed to detect GFLV RNA2 was the most sensitive technique (1E–04). It was 10 times more sensitive than IC-RT-PCR which was also ten times more sensitive than DAS-ELISA, except for variant IC-MaA8193.

**Table 4:** Sensitivity of three diagnostic techniques for five GFLV isolates.

		IC-RT-PCR		IC-RT-qPCR	
GFLV isolates	DAS-ELISA	RNA1	RNA2	RNA1	RNA2
B844	1E–02	1E–03	1E–03	1E–04 (38.6}	1E–04 (36.5)
F13	1E–02	1E–03	1E–03	1E–03 (37.3)	1E–04 (36.2)
GHu	1E–02	1E–03	1E–04	1E–04 (39.5}	1E–04 (36.8)
C01 (A17b)	1E–02	1E–03	1E–03	1E–03 (37.9)	1E–04 (34.3)
MaA8193	1E–03	1E–01	1E–03	1E–04 (37.0)	1E–04 (35.2)

*Sensitivity is expressed as the last dilution of crude leaf sap for which a positive reaction is obtained, with the first dilution being 0.5 g of frozen tissue in 5 mL of DAS-ELISA grinding buffer. For IC-RT-qPCR, C_t_-values at which the virus is detected are indicated in parenthesis.*

**FIGURE 3:**
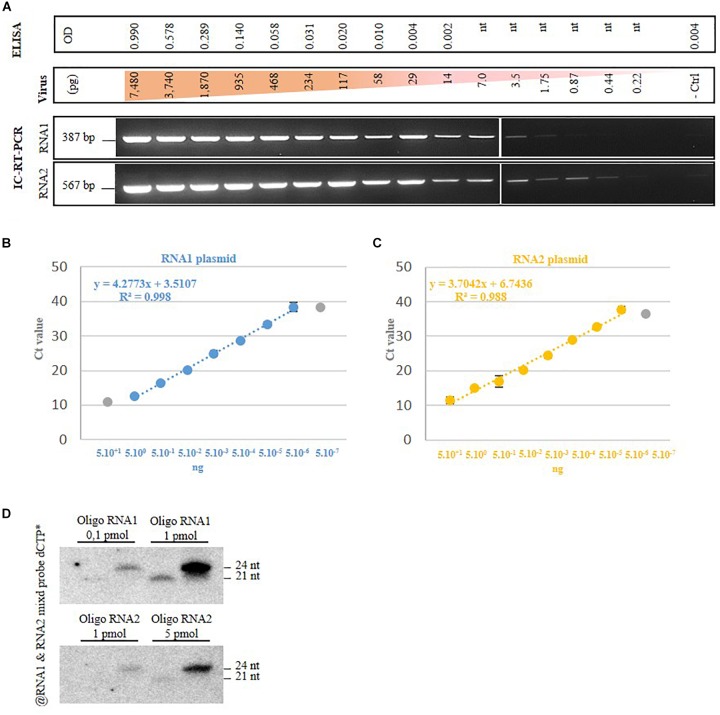
Grapevine fanleaf virus detection limit of four diagnostic techniques. **(A)** DAS-ELISA and IC-RT-PCR with serial dilutions of purified virion, with nt, not tested. - ctrl, healthy plant. **(B)** RT-qPCR with dilution of plasmids carrying GFLV RNA1 and RNA2 cDNAs and **(C)** vsiRNA blots with dilution of RNA-based oligonucleotides.

Then we assessed the detection limit for each method selected in this study. For the antibody-based detection techniques (DAS-ELISA and IC-RT-PCR), we started from a known quantity of a purified GFLV strain GHu preparation from which serial dilutions were performed from 7,480 to 0.22 pg. For DAS-ELISA, the detection limit was set at 117 pg of virus (Figure 3A, higher part of the panel), which corresponded to the dilution above the background set at 2.5× the optical density (OD_405nm_) of the negative control. From the same GFLV purification, 1.75 pg of virus was detected by IC-RT-PCR when using RNA1 specific primers and 0.44 pg of GFLV was detected when using RNA2 specific primers (Figure 3A, lower panel). This result indicated that IC-RT-PCR is 60–250x more sensitive than DAS-ELISA for GFLV diagnosis. In order to define the RT-qPCR-based detection limit, we used known amounts of plasmids carrying cDNAs of GFLV RNA1 or RNA2, from which serial dilutions were made. The last dilution detected by qPCR was 5 fg for both plasmids which corresponds to 480 molecules of RNA1 and 780 molecules of RNA2 (Figure 3B). To estimate the vsiRNA blot detection limit, small RNAs of 21 and 24 nt corresponding to conserved sequences within the five probes used for hybridization were designed. As low as 0.1 pmol of siRNA RNA1 was detected by vsiRNA blot (Figure 3C).

### Field Samples Study

To compare the different techniques in a real detection setting, twenty samples from two GFLV-infected vineyard plots located in the Champagne region of France were evaluated. Thirteen out of the 20 samples were positive for GFLV by DAS-ELISA while seven samples were negative (Table 5). Viral quantity ranged from 1,497 to 24,712 ng of virion per gram of fresh leaf material in infected samples. An immuno-capture assay followed by RT-PCR confirmed the aforementioned results, with the same 13 samples testing positive for GFLV and the same seven samples testing negative using either RNA1 or RNA2 specific primers (Table 5 and Supplementary Figure S2A). The same 13 samples were confirmed positive for GFLV by RT-qPCR, except sample 1 for which RNA1 was below the detection level (Table 5), while RNA2 was unquestionably detected in that particular sample. All seven samples found GFLV negative by the two antibody-based techniques were confirmed negative by RT-qPCR by using primers for both genomic RNAs. Northern blot for vsiRNA validated the results obtained by the other detection methods, with the same 13 samples displaying vsiRNA, while seven did not (Table 5 and Supplementary Figure S2B).

**Table 5:** Grapevine fanleaf virus detection in 20 vineyard samples by five techniques.

		Sample names																			
		Pa1	Pa2	Pa3	Pa4	Pa5	Pa6	Pa7	Pa8	Pa9	Pa10	Py11	Py12	Py13	Py14	Py15	Py16	Py17	Py18	Py19	Py20
DAS-ELISA (ng/g)		3,104	6,782	–	7,004	–	1,497	–	4,531	–	4,372	–	6,581	–	23,654	4,425	–	24,712	18,077	4,393	6,518
IC-RT-PCR	RNA1	+	+	–	+	–	+	–	+	–	+	–	+	–	+	+	–	+	+	+	+
	RNA2	+	+	–	+	–	+	–	+	–	+	–	+	–	+	+	–	+	+	+	+
RT-qPCR (mol/ng)	RNA1	–	94,258	–	115,494	–	68,575	–	12,321	–	10,944	–	60,934	–	59,227	52,485	–	13,132	11,489	4,776	16,898
	RNA2	40,369	200,184	–	267,744	–	25,6424	–	26,717	–	91,501	–	94,271	–	193,214	174,371	–	219,388	159,640	166,384	124,855
vsiRNA blot		+	+	–	+	–	+	–	+	–	+	–	+	–	+	+	–	+	+	+	+
RNAseq	RNA1	363	925	–	669	–	382	–	1,428	–	1,060	–	524	–	922	618	–	1,181	853	528	425
(RPKM)	RNA2	668	2,097	–	1,249	–	1,340	–	3,709	–	2,268	–	1,313	–	2,164	1,847	–	2,250	2,636	1,152	682
	RNA3	–	925	–	900	–	335	–	–	–	–	–	–	–	–	–	–	1,993	–	–	–

*DAS-ELISA: ng of virions per gram of leaves (ng/g); RT-qPCR: number of RNA molecule per ng of total RNA (mol/ng) and RNAseq: reads per kilobase Million (RPKM). For IC-RT-PCR and vsiRNA, see Supplementary Figure S2. Negative samples (-) with a reaction below the detection limit (117 pg for ELISA, 480 RNA1 and 780 RNA2 molecules for RT-qPCR, no visible amplicons in IC-RT-PCR nor vsiRNA on the blots) and a negative outcome following our intra-lane contamination protocol for RNAseq are indicated.*

We finally performed RNAseq with total RNAs used in RT-qPCR. The twenty samples were multiplexed in a single RNAseq lane and an average of about 34 million raw reads (ranging from 31 to 44 M, Supplementary Table S3) was obtained per sample. Interestingly, all samples displayed reads corresponding to the GFLV genome, ranging from 99 to 379,611 reads and 127 to 506,907 reads mapping to the GFLV consensus RNA1 and RNA2 sequences, respectively (Supplementary Table S6). However, treating the data with our intra-lane contamination detection protocol, the seven samples that were found negative using the previously ran methodologies were negative for GFLV, showing consistency among all the results (Table 5). However, for GFLV quantification, no correlation was found among diagnostic techniques, as expected (Supplementary Table S8).

From the RNAseq libraries, 26 GFLV RNA1, 18 GFLV RNA2, and three GFLV RNA3 complete to near complete (*i.e.*, covering at the minimum the open reading frame) sequences were *de novo* assembled (Supplementary Table S7), underlying the fact that 10 grapevines were co-infected with more than one RNA1 molecule and five of them with two RNA2 molecules.

### Additional Information on the Virome in Two Selected Vineyard Plots in the Champagne Region of France

From the RNAseq datasets, information on the presence of viroids and other poly-A-tailed virus genomes was obtained (Supplementary Table S7). GRSPaV, HSVd and GYSVd1 were readily detected and assembled from all samples. A majority of samples (70%) was also infected with grapevine fleck virus (GFkV). Grapevine rupestris vein feathering virus (GRVFV) and grapevine Syrah virus 1 (GSyV-1) were also easily detected. Sample Pa10 was infected with grapevine Pinot gris virus (GPGV). Additionally, the presence of grapevine leafroll-associated virus 2 (GLRaV-2) with a non-poly-A-tailed genome was unquestionably detected in samples Pa4, Pa8, Py14, and Py17, although the full-length viral genome was not obtained. The presence of these viruses was corroborated via RT-PCR: GPGV and GRVFV in sample Pa10, GSyV-1 in sample Pa5 and GLRaV2 in Py14 (data not shown). These results confirm that grapevine is rarely infected by a single virus species, or even a single variant of that species, but rather by multiple viruses in a vineyard. Only samples Pa3 and Py13 were only co-infected with GRSPaV and viroids that are present in most grapevines worldwide and are considered as part of the ‘microbiome’ of grapevine (Saldarelli et al., 2017). All the other samples were super-infected with at least one (samples Pa7, Pa9, Py16, and Py19) and up to five different viral species (sample Py17). Nonetheless, the virus count might not be final, knowing that RNAseq does not provide an exhaustive view of the virome.

## Discussion

In this work we compared RNAseq with semi-quantitative DAS-ELISA, IC-RT-PCR, RT-qPCR and vsiRNA blot for the detection of GFLV. Our results showed that GFLV is readily detected by the five diagnostic methodologies (Figure 1). These techniques covered the viral diversity exemplified by five GFLV strains (Supplementary Figure S1 and Table 4). Diagnostic results were consistent across the five methods, from the very cheap and dependable DAS-ELISA to the massive parallel sequencing strategy offered by RNAseq. The reliability of DAS-ELISA was reported in previous studies with only rare cases of GFLV variants not being detected (Liebenberg et al., 2009; Bruisson et al., 2017). Our findings also confirmed that IC-RT-qPCR has a better detection sensitivity compared to IC-RT-PCR which was also better than that of DAS-ELISA (Table 4). Interestingly, detecting the presence of GFLV was more sensitive when targeting RNA2 compared to RNA1. Likewise, when dealing with RNAseq datasets, RPKMs (which reflect viral accumulation) were always higher for RNA2 compared to RNA1 sequences (Table 5). This higher accumulation of RNA2 was previously described from the estimation of the quantity of RNA species from purified GFLV particles (Pinck et al., 1988).

As expected, no correlation in GFLV quantification was observed between the different diagnostic techniques (Supplementary Table S8). What was more startling was that no correlation in viral quantification was found between techniques that identify the same target, for example total RNA by RT-qPCR and RNAseq. This could be due to an inherent bias of each method such as the use of RPKM that normalize the coverage/quantification along the whole molecule for RNAseq compared to the involvement of anchored primers in the RT-qPCR method, amplifying only one small targeted portion of the viral genome. The use of specific primers might also prevent the amplification of all variants by RT-qPCR due to mismatches. However, correlations were found when comparing GFLV RNA1 and RNA2 quantification by the same detection technique, strongly supporting the fact that both RNAs are necessary for a successful infection to take place and probably at a steady equilibrium, with a RNA2/RNA1 ratio = 2.37 ± 0.16 according to our RNAseq dataset. This ratio was quite similar to the 2.33 ± 0.06 ratio from one of our previous metagenomics study (Hily et al., 2018c). This result reinforces the need to target RNA2 for a more efficient GFLV detection by molecular techniques.

High throughput sequencing offers unparalleled amount of sequence information from a single run. From this work using either RNAseq or smallRNAseq libraries, we learned that the number of reads or the genome coverage are not sufficient to proclaim a sample positive for a virus (Table 1). After a direct mapping analysis from the RNAseq datasets, both HRC and LRC samples displayed over 80% coverage of the GFLV genome. By performing a simple *de novo* assembly from reads mapping to GFLV-consensus sequences in the LRC samples, not a single complete GFLV sequence was obtained but rather a number of short contigs that matched complete GFLV genomes *de novo* assembled from HRC samples present in the same sequencing lane. From these observations, we developed a bioinformatics protocol to remove highly identical reads from different samples within the same sequencing lane (Figure 2 and Supplementary Data Sheet 1), allowing for a discrimination between potential low viral titer infection and intra-lane cross-contamination, also known as ‘index misassignment’ or ‘index hopping’ (Kircher et al., 2012). Such contamination arises when multiple libraries are pooled in a single lane and sequenced simultaneously. To address this issue, a unique sequence (called index) per sample is added to individual DNA fragments during a library preparation. Index hopping occurs post-sequencing and before final data analysis during a process called ‘demultiplexing.’ Levels of index hooping are different depending on the indexes being used^3^. This step consists in identifying all reads by sorting them computationally to its correct library. While index hopping is inevitable when multiplexing samples, few practices offer mitigation options, with the best of them consisting in the use of a unique dual indexing combinations (Kircher et al., 2012). However, we clearly show that the use of dual indexing is not sufficient to completely abolish such index hopping (see results from Lane 1). This is why other options to identify and evaluate contaminations are needed. Our contamination evaluation protocol is quite robust, however, it will not discriminate contaminations generated prior to sequencing, for example, contamination produced during nucleic acid extraction, through the addition of indexes and PCR steps. Furthermore, one main drawback of our procedure is when a LRC sample is co-infected with a virus at low titer and a virus with the same (or very close) genome sequence to the HRC one(s) pooled in the same sequencing lane. In that particular case, it would be technically impossible to discriminate between contamination and low viral titer.

With this study, we confirmed that more than just a detection tool, these massively paralleled sequencing techniques can detect and discriminate, within a sample and from a single run, one or more variants of the same virus or of different viral species (Table 3, Supplementary Figure S3 and Supplementary Table S7; Beuve et al., 2018; Hily et al., 2018a,c). Such feat is not possible using traditional detection technics unless, for example, the PCR-based techniques have been beforehand specially designed for this purpose, requiring additional tune-up and development prior to the analysis. Other information can be mined when using HTS, focusing not only on a single virus but describing the full virome of a plant. This terminology, describing the collection of nucleic acid that make up the viral community within a sample, takes all its value when dealing with a perennial plant such as grapevine. In this work, we confirmed that the virome of a vineyard is often more complex than previously thought, with grapevines being simultaneously infected by many viral species (Table 3 and Supplementary Table S7; Coetzee et al., 2010; Eichmeier et al., 2016; Fajardo et al., 2017; Beuve et al., 2018). HTS studies previously revealed the presence of many mycoviruses (Al Rwahnih et al., 2011; Espach et al., 2012)^4^, as well as viruses with unknown host (Hily et al., 2018b) but associated with grapevine. All aforementioned reports are describing the complex interaction between microbial communities within a particular ecosystem, ultimately questioning the concept and the definition of a ‘healthy’ vine. Here, we confirm the presence of several virus species of the family *Tymoviridae* such as GFkV, GSyV-1, and GRVFV, in samples from two vineyards in the Champagne region of France. These might ultimately be considered as GRSPaV, HSVd and GYSVd to be part of the ‘background’ virome of grapevine (Saldarelli et al., 2017).

Our data highlighted some advantages and drawbacks of RNAseq and smallRNAseq libraries. For example, the latter offers a wider viral detection panel, not restricted to poly-A-tailed viruses (Table 3). On the other hand, *de novo* assembly and full-length genome sequence recovery was more difficult than from RNAseq libraries. Often some viral genomes were not fully recovered from smallRNAseq datasets, with small gaps along the genome (data not shown). This was also previously reported for other sequencing techniques (Beuve et al., 2018). Except for GFkV or other viruses with a cytosine-rich genome, RNAseq provided data that can yield complete viral genomes as a result of a straightforward *de novo* assembly protocol. Moreover, viral genetic diversity was better defined using RNAseq. For example, within our parameters (quality and sequencing depth), little to no difference in reads mapping were observed between GFLV-consensus and *de novo* assembled sequences from our RNAseq datasets. This indicated that using a consensus genome might be the best approach to recover a maximum viral diversity within a sample even when dealing with a highly divergent virus such as GFLV. On the other hand, read numbers nearly tripled when dealing with smallRNAseq data. More work in parameter setting is needed to easily detect the full genetic diversity within a sample when using smallRNAseq libraries. Another advantage that HTS offers compared to traditional methods is the possibility to go back to datasets which can be considered as ‘evergreen’ and can be analyzed endlessly. This is particularly advantageous because new viruses are detected almost monthly.

All detection techniques were in agreement for the characterization of the presence or absence of GFLV in samples from a grapevine virus collection or naturally infected vineyards. We can conclude that (i) DAS-ELISA is a robust and reliable method detecting as low as 117 pg of virion, (ii) Molecular approaches are, as expected, more sensitive than serological methods, regardless of the isolate tested with IC-RT-qPCR allowing the detection of GFLV as low as 0.44 pg of virions when targeting RNA2, (iii) SmallRNAseq libraries are useful for an exhaustive definition of the sanitary status of a sample, (iv) RNAseq is the method of choice for an easy detection of distinct GFLV variants, and (v) a suitable protocol discriminating between contamination and the potential low GFLV infection level needs to be implemented for RNAseq and small RNAseq datasets to confidently decide on the infectious status of grapevine samples.

## Data Availability

The raw data supporting the conclusions of this manuscript will be made available by the authors to any qualified researcher upon request.

## Author Contributions

EV, OL, and J-MH designed the experiments. EV, SG, VK, and J-MH performed the experiments. EV, OL, and J-MH wrote the paper. All authors contributed to the manuscript revision, read and approved the submitted version.

## Conflict of Interest Statement

The authors declare that the research was conducted in the absence of any commercial or financial relationships that could be construed as a potential conflict of interest.

## Footnotes

1 http://www.plantgdb.org/XGDB/phplib/download.php?GDB=Vv (Genoscope 12x, last visited March 2018)

2 http://blast.ncbi.nlm.nih.gov/Blast.cgi (last visited March 2018)

3 https://www.illumina.com/content/dam/illumina-marketing/documents/products/whitepapers/index-hopping-white-paper-770-2017-004.pdf (last visited May 2018)

4 http://www.wineland.co.za/virus-diversity-associated-grapevine-leafroll-disease/ (last visited May 2018)
