# Supplementary figures and images for: Comparison of Serological and Molecular Methods With High-Throughput Sequencing for the Detection and Quantification of Grapevine Fanleaf Virus in Vineyard Samples
Source: Front Microbiol. 2018 Nov 22;9:2726. doi: 10.3389/fmicb.2018.02726 (PMC6262039; doi:10.3389/fmicb.2018.02726)

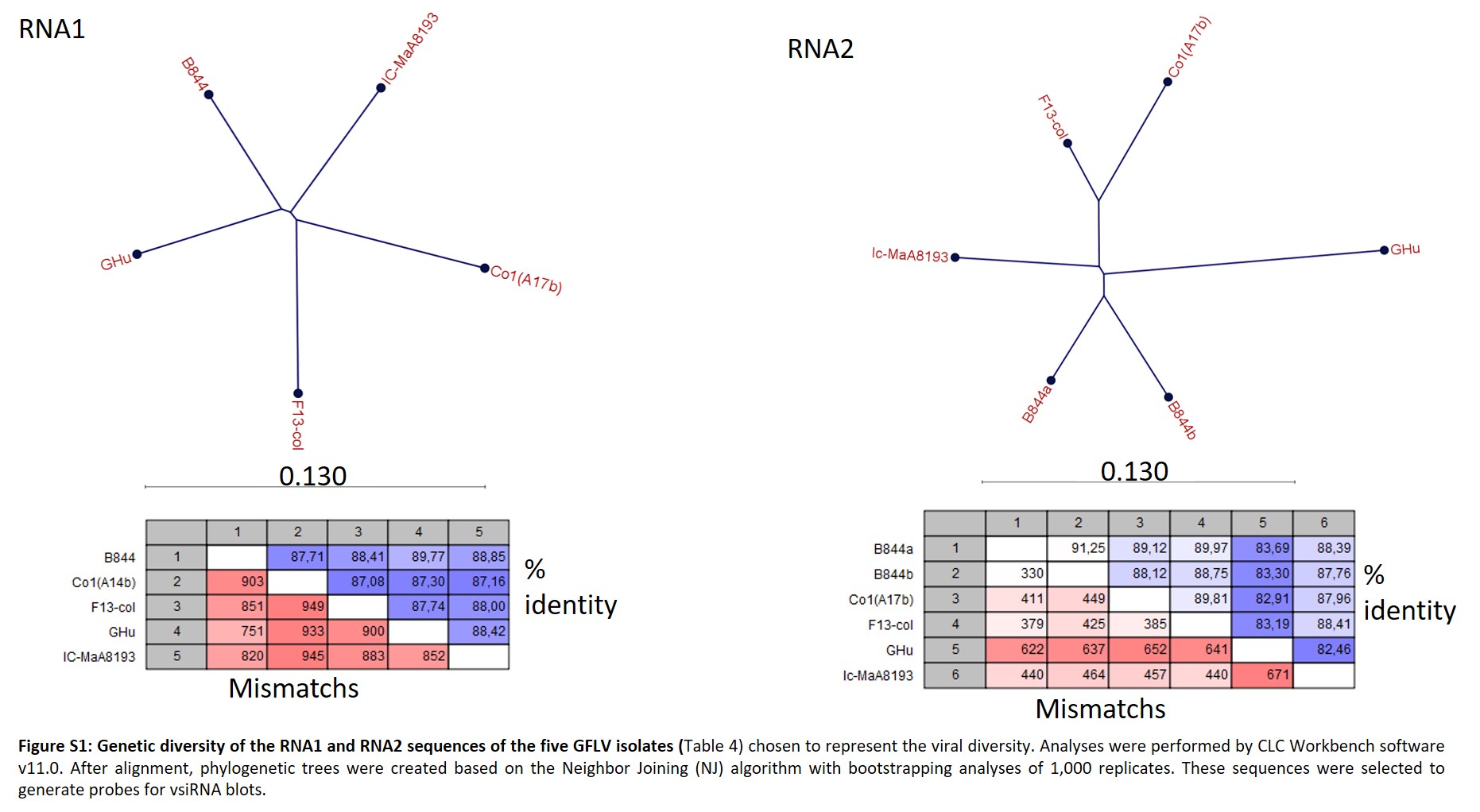

Supplement: Supplementary file 1 [file Image_1.JPEG]

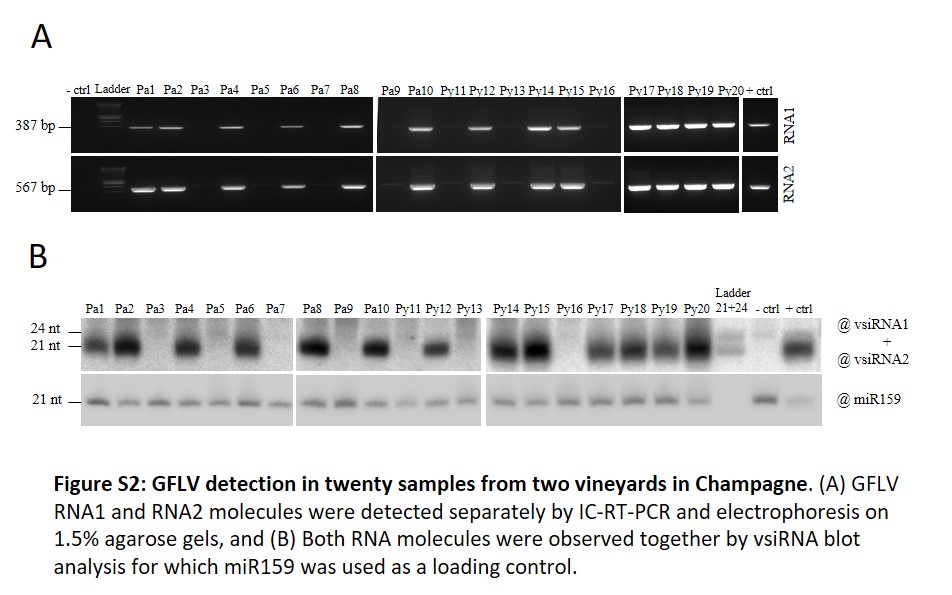

Supplement: Supplementary file 2 [file Image_2.JPEG]

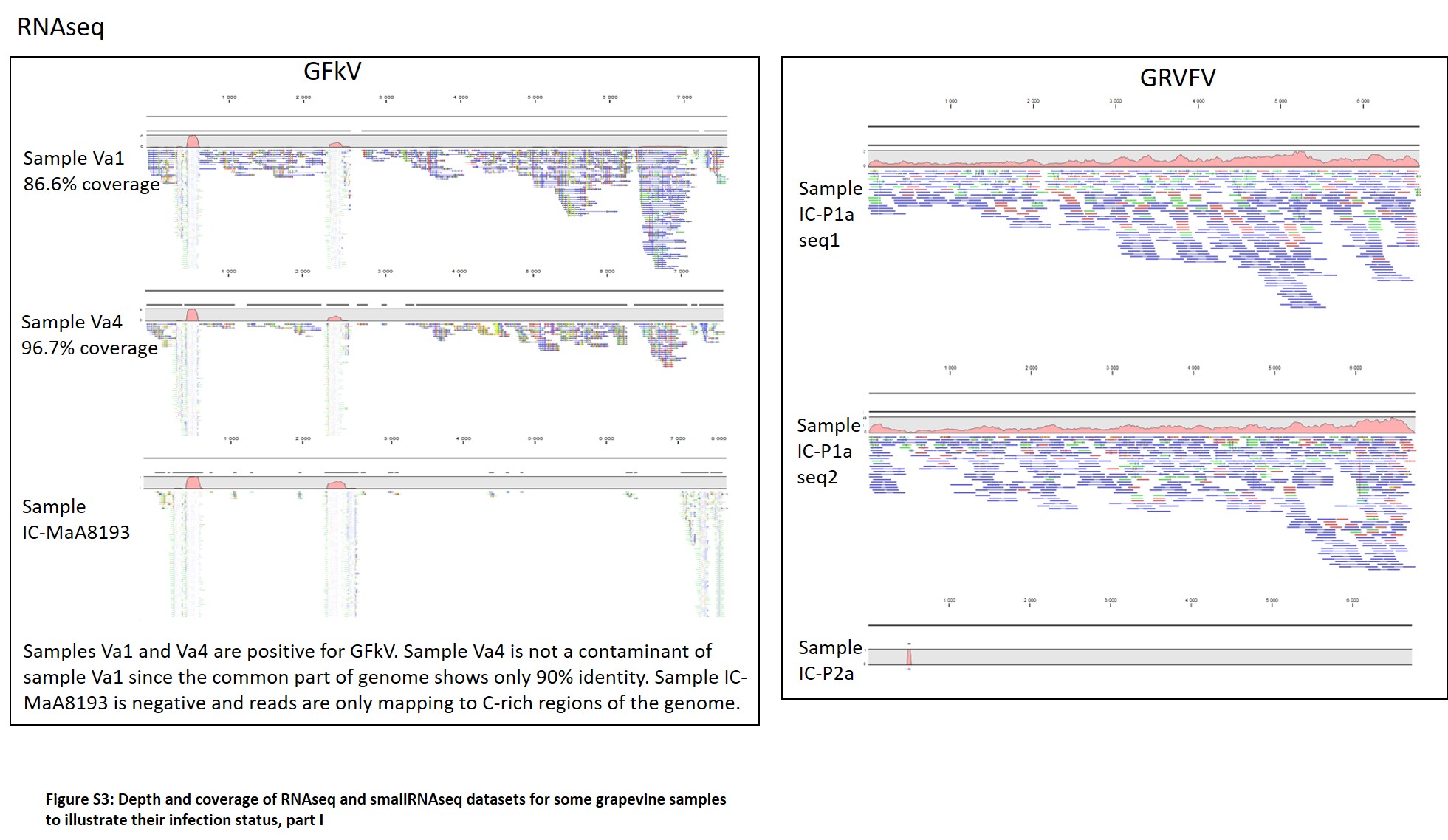

Supplement: Supplementary file 3 [file Image_3.JPEG]

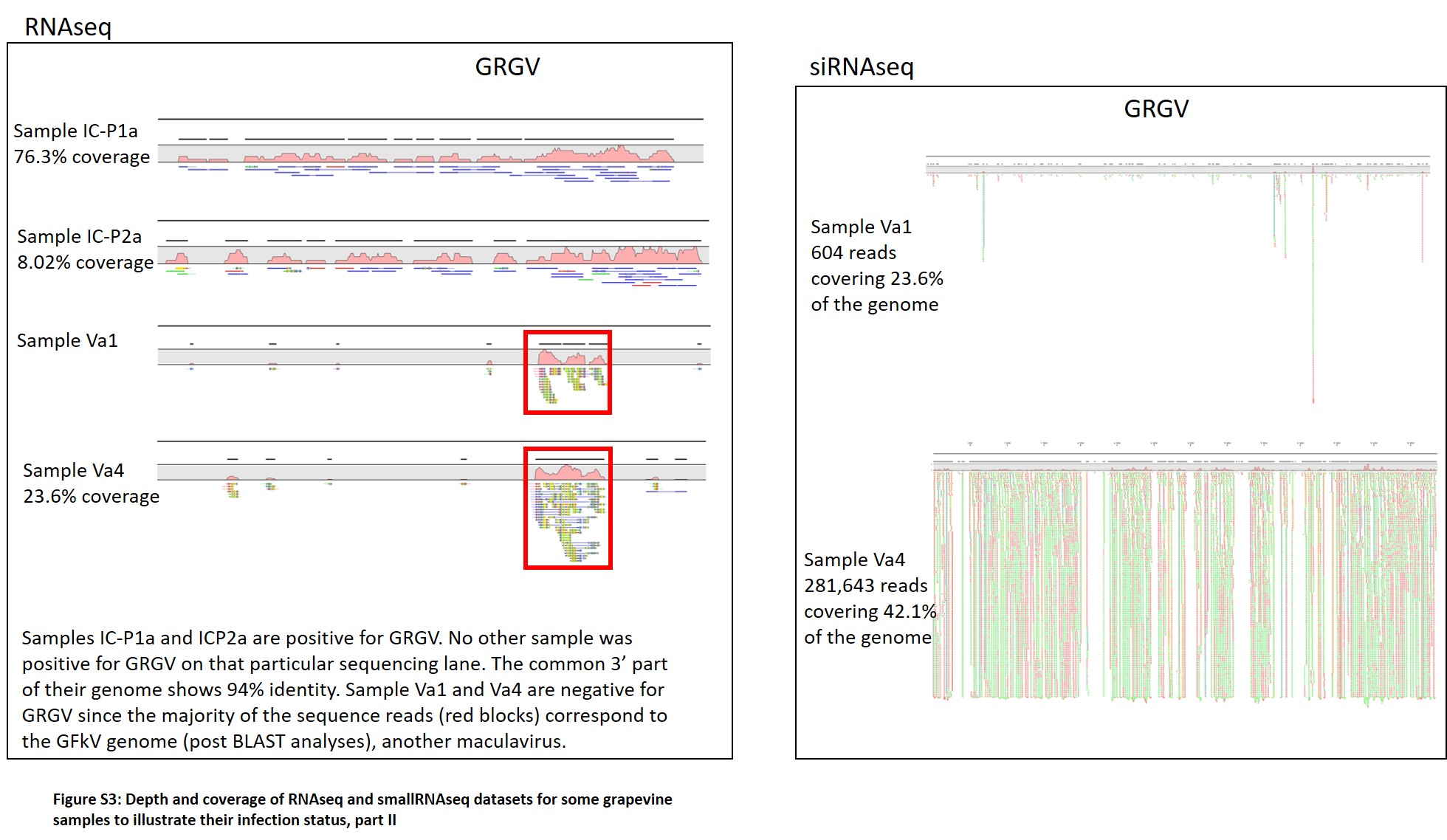

Supplement: Supplementary file 4 [file Image_4.JPEG]

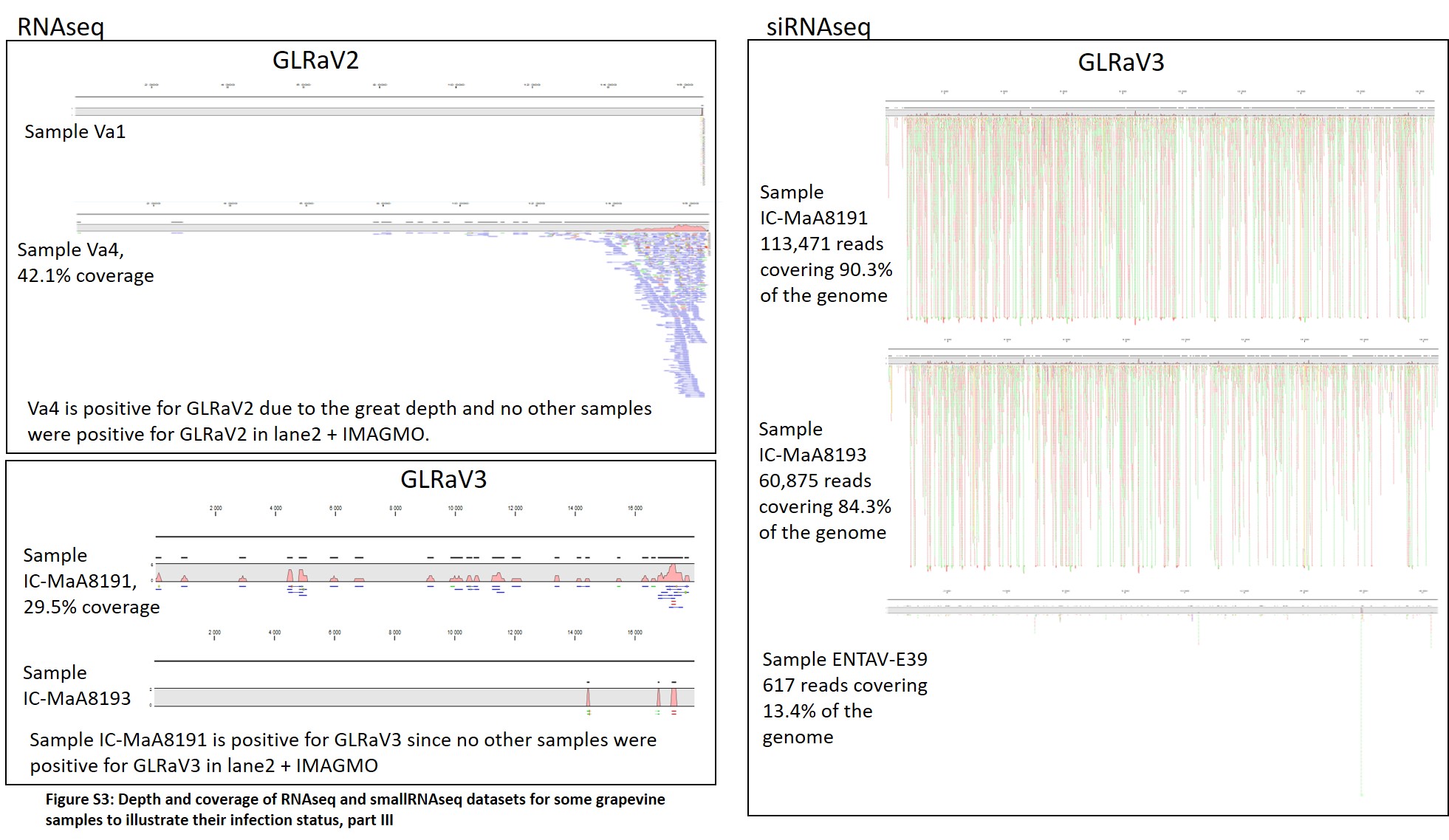

Supplement: Supplementary file 5 [file Image_5.JPEG]
