# Supplementary material for: Comparison of Serological and Molecular Methods With High-Throughput Sequencing for the Detection and Quantification of Grapevine Fanleaf Virus in Vineyard Samples
Source: Front Microbiol. 2018 Nov 22;9:2726. doi: 10.3389/fmicb.2018.02726 (PMC6262039; doi:10.3389/fmicb.2018.02726)
Supplement: Supplementary file 6 [file Data_Sheet_1.docx]

**Supplemental protocol: GFLV intra-lane contamination detection from RNAseq libraries.**

This protocol was designed to distinguish between intra-lane contamination due to ‘index hopping’ and low viral titer in a sample. For this step-by-step protocol, we present the case of sample Pa9. This protocol was completed using the CLC Genomics Workbench v.11.0 software (Qiagen).

1- Take the pool of clean reads from your sample of choice and map it to the reference sequences. Here, 33,606,334 reads were mapped to GFLV consensus sequences.


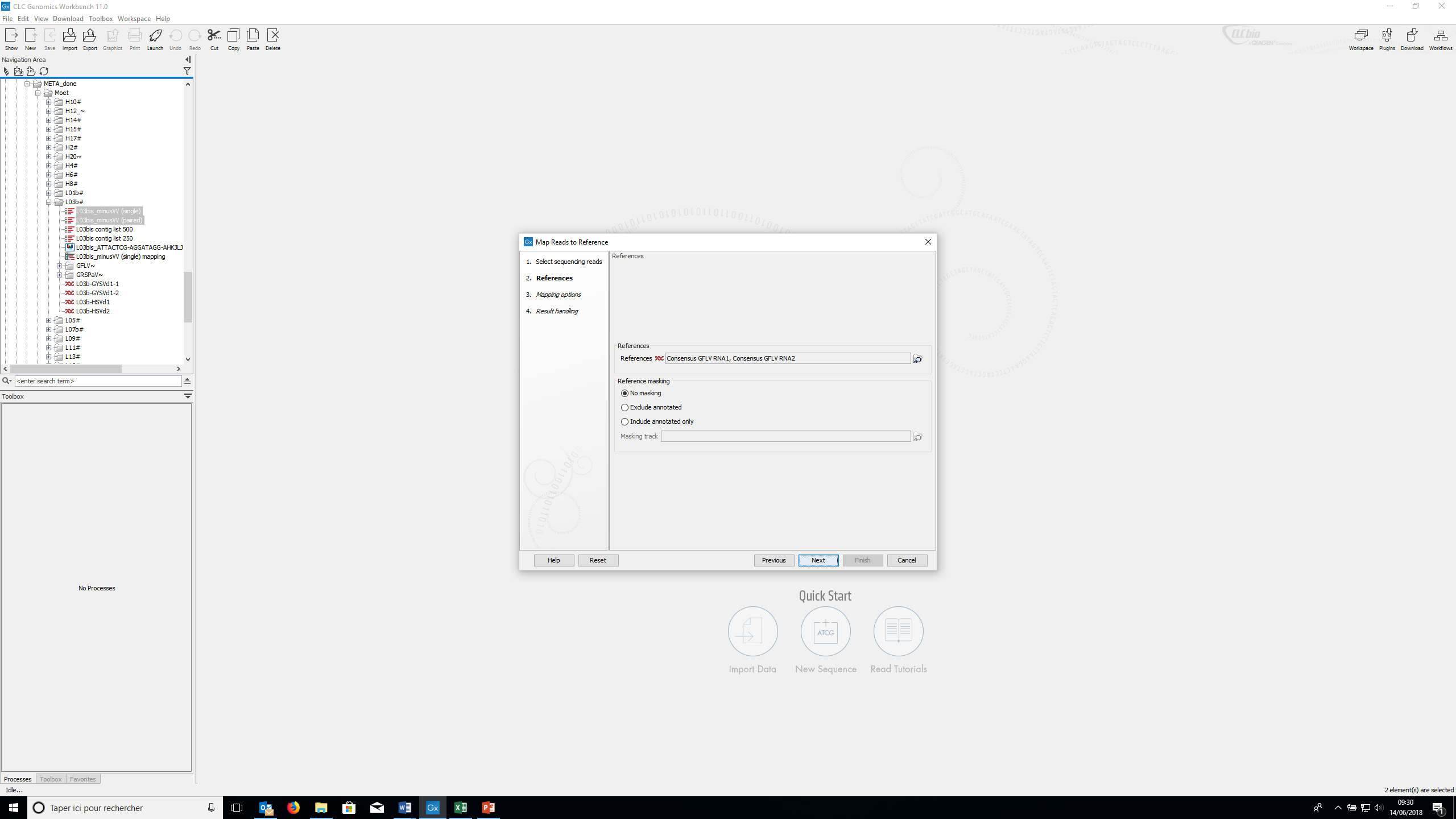


2- Use relaxed parameter to recover a maximum of the GFLV genetic diversity.Here, 0.5 was used as length fraction and 0.7 as similarity fraction.


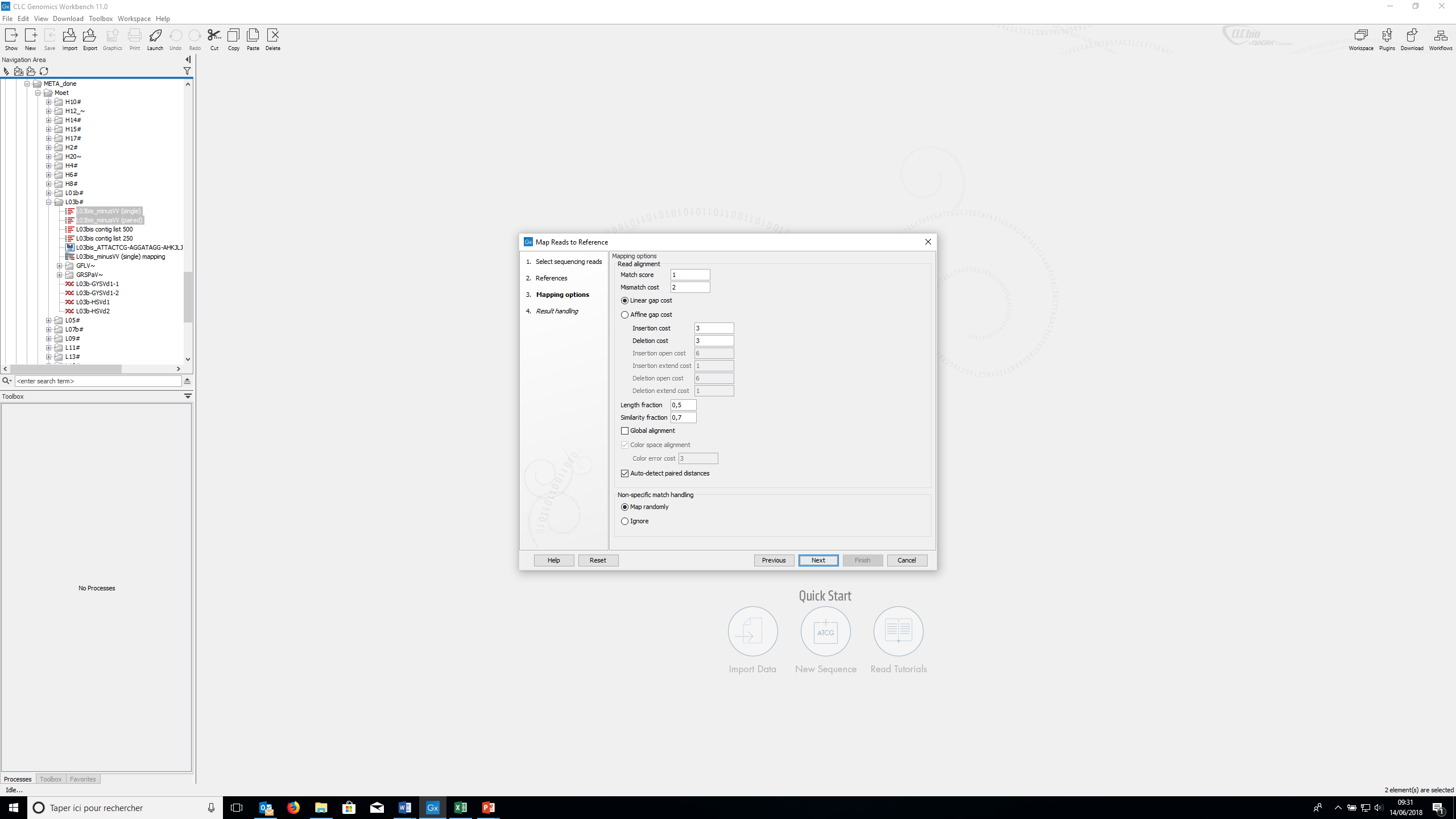


3- Post-mapping results and Visualization of the reads dispersion along the reference sequence. Here we chose to focus on the RNA2 sequence. This corresponds to step in Figure 2.

2


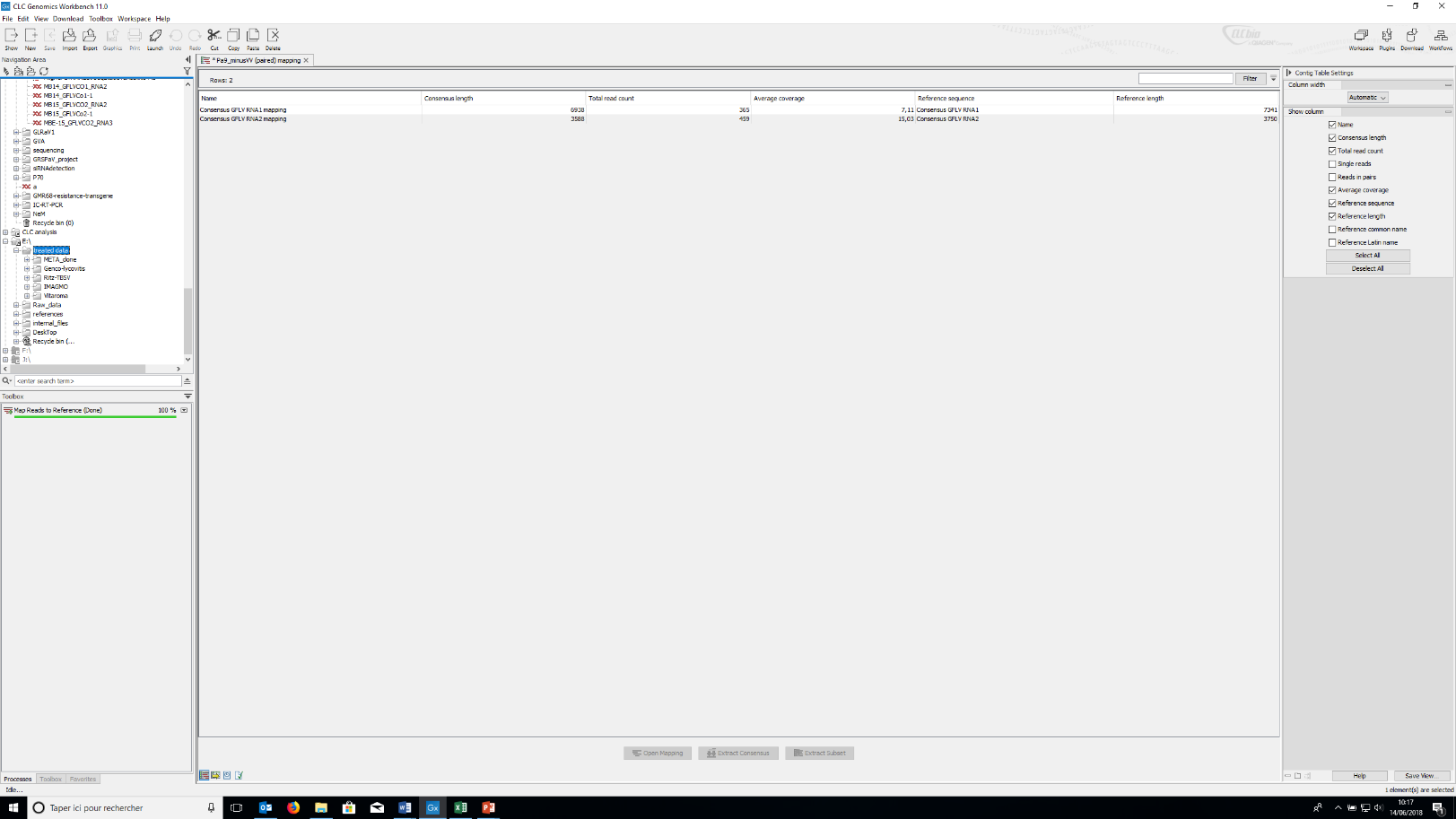


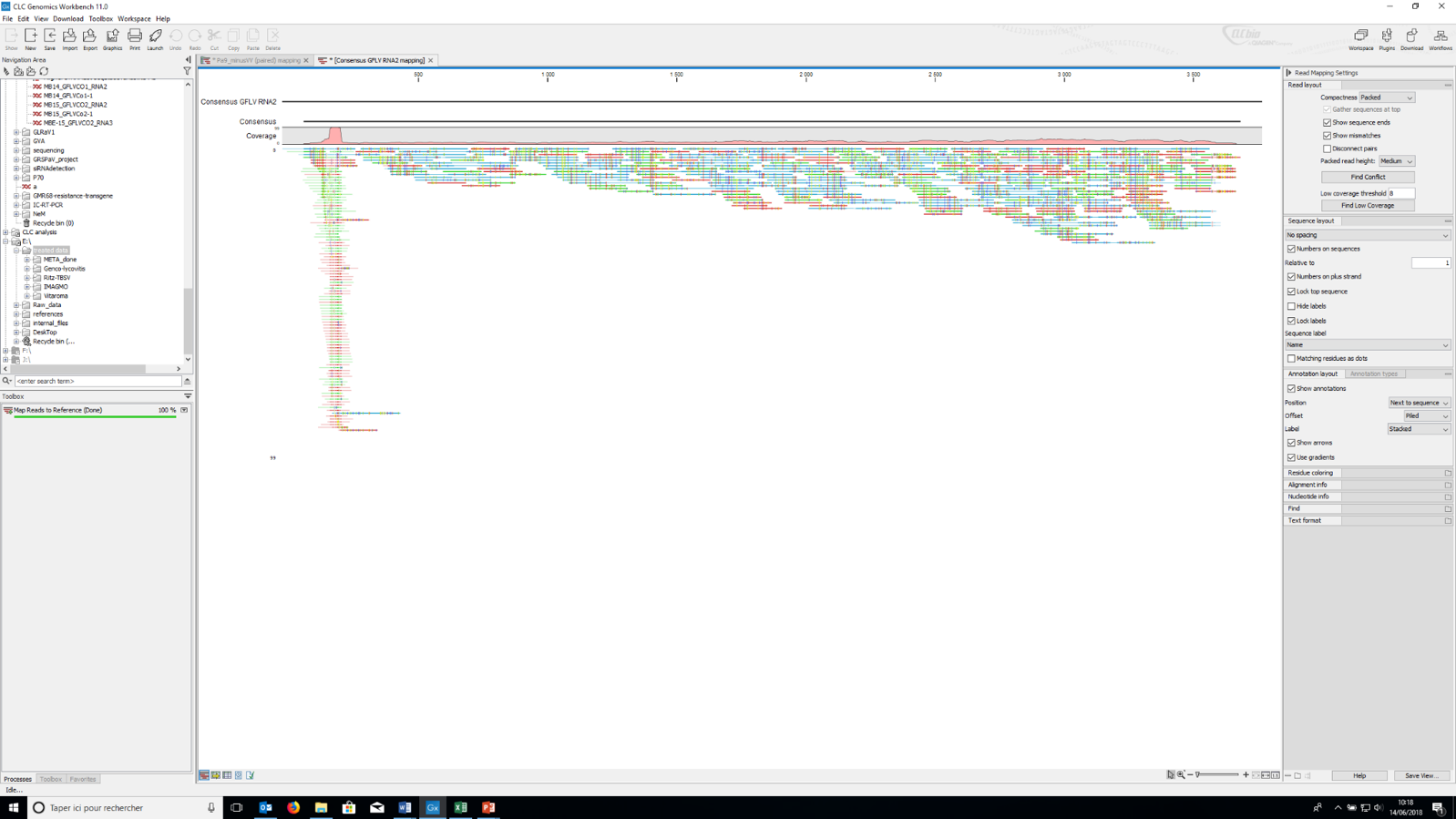


For this sample, 459 reads map to the GFLV RNA2-consensus sequence and cover 96% of the sequence. Paired reads are in blue, while singletons are in red and green. On the left hand-side, there is an over-mapped region of the genome.


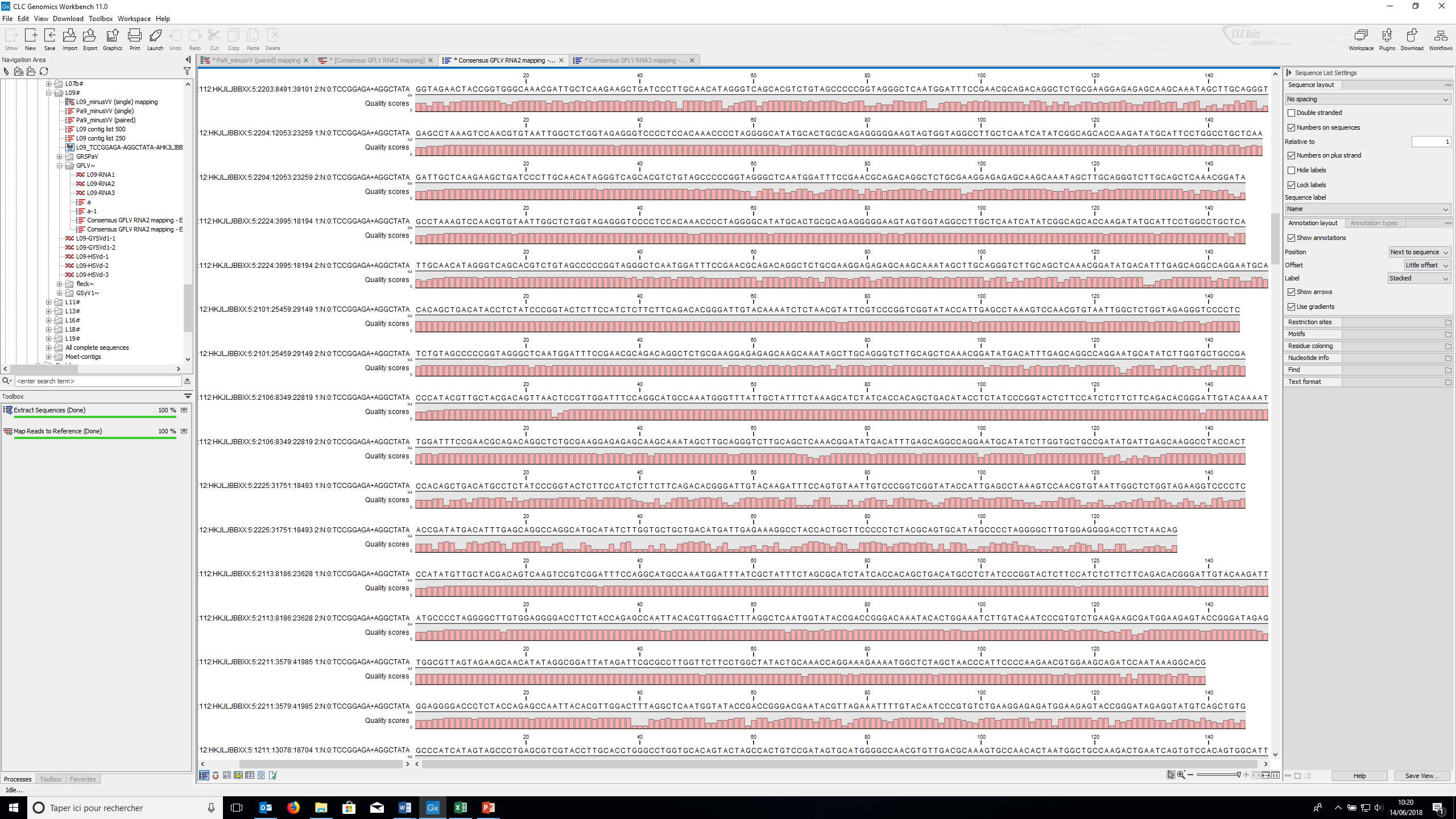
4- Save the reads that mapped to the reference sequence for downstream analyses. In this case, we saved the 459 reads, consisting of paired reads and singletons.


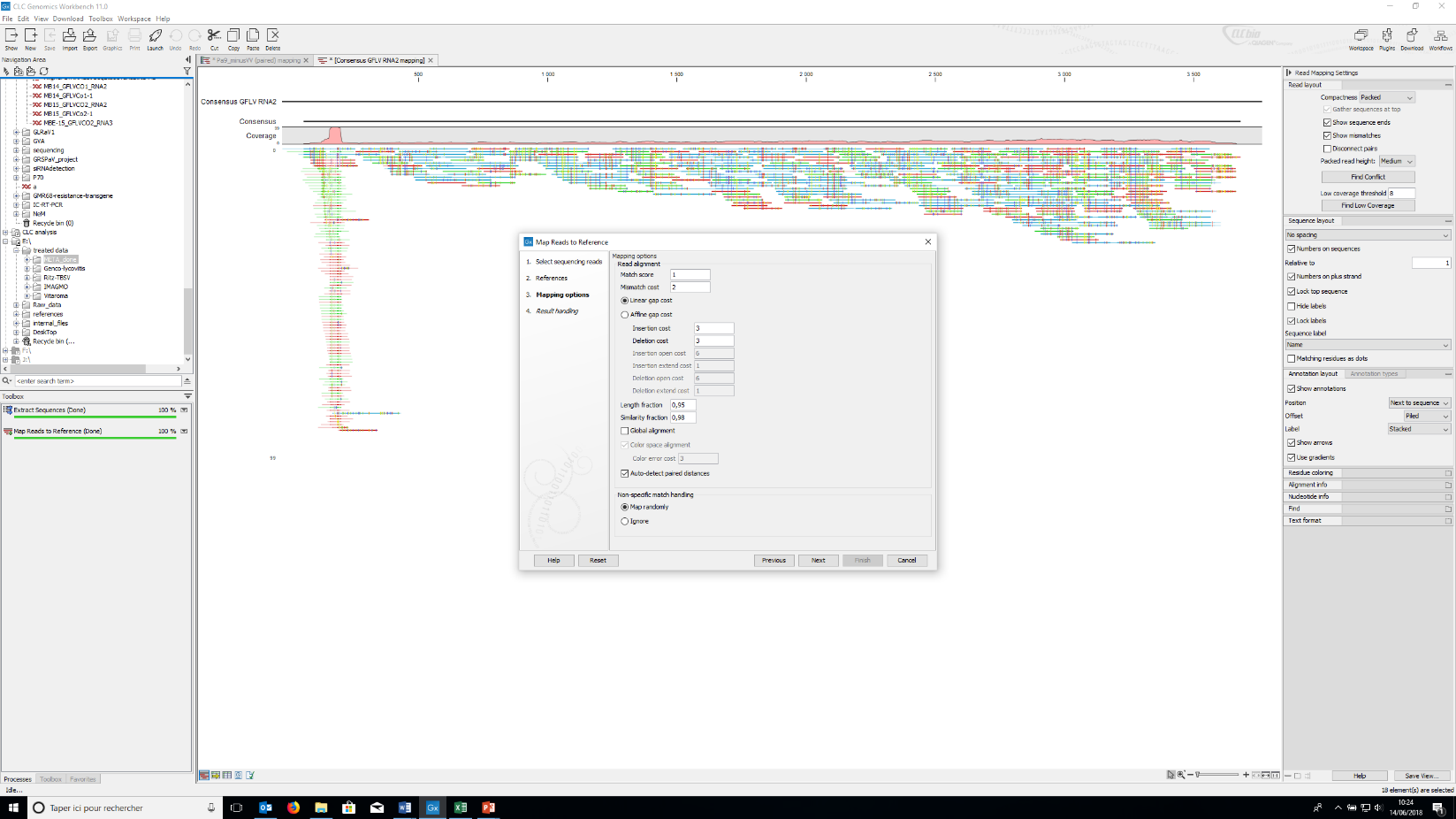
5- Map all recovered reads to all GFLV RNA2 sequences *de novo* assembled from samples sequenced from the same sequencing lane as your sample. In our case, we assembled 18 RNA2 sequences from 13 samples using very stringent parameters of 0.95 for length fraction and 0.98 for similarity fraction.

6- Prior to performing the mapping, do not forget to check the ‘collect un-mapped reads’ box.


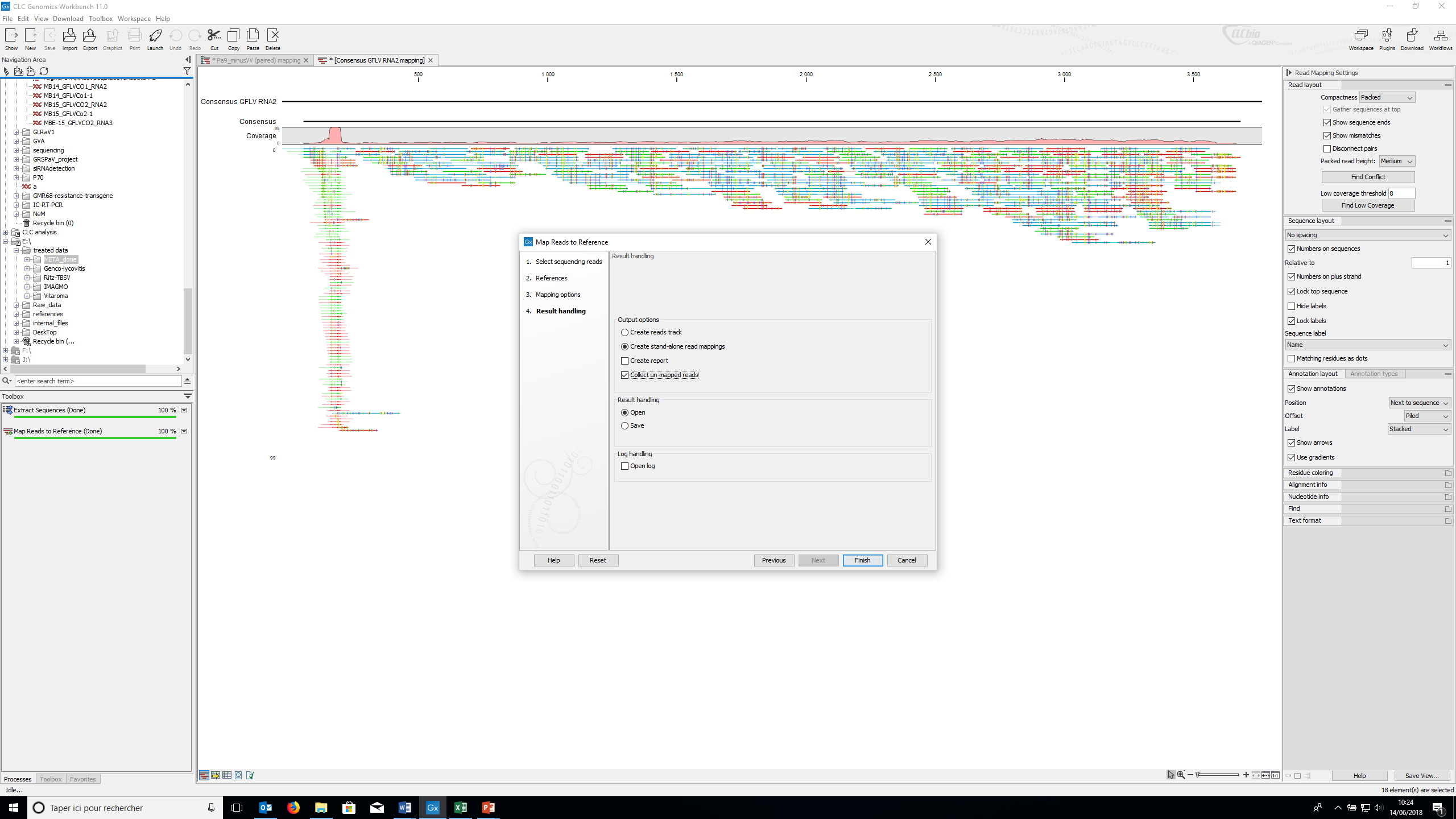


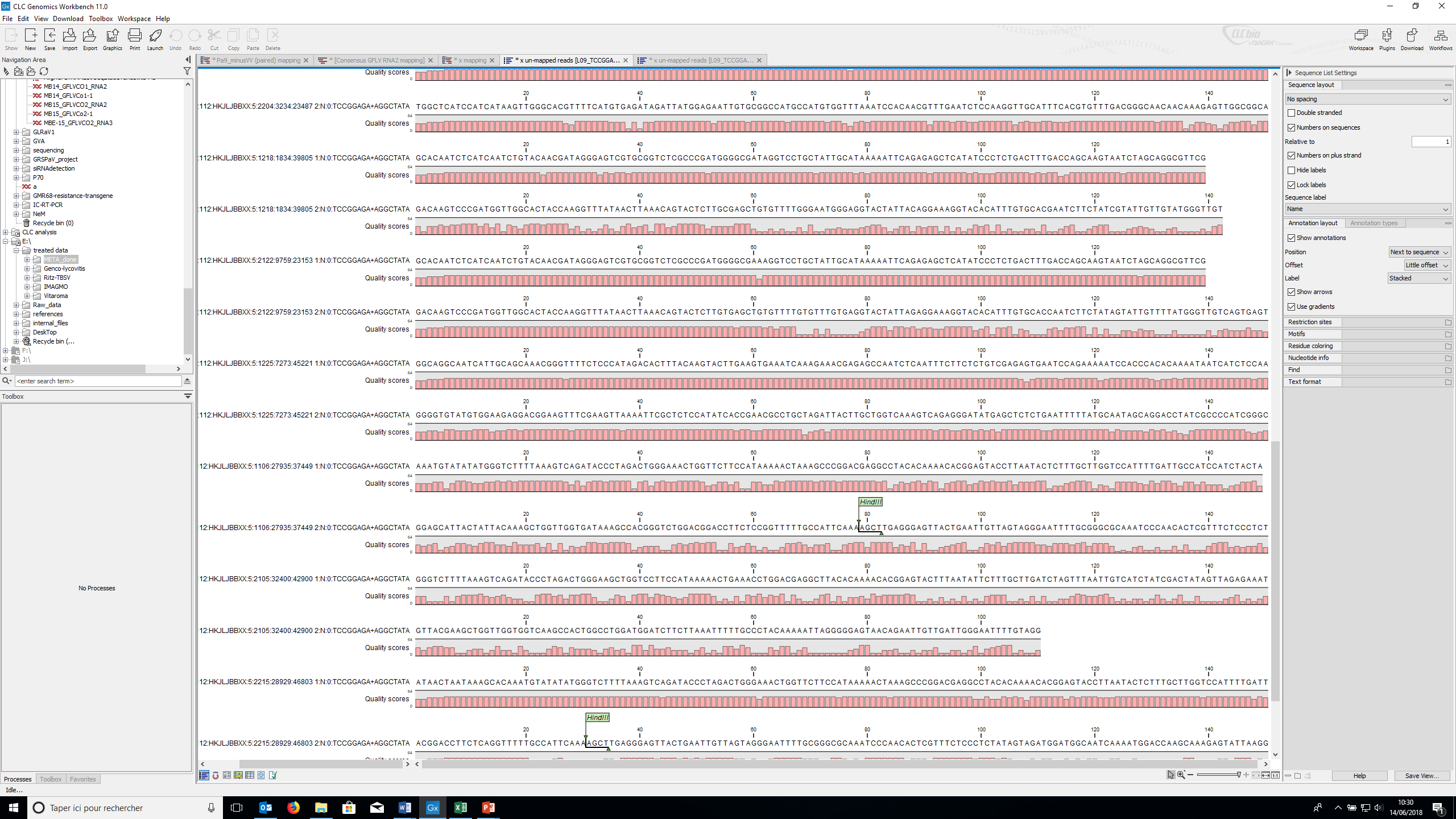
7- Save the ‘un-mapped’ reads, consisting of paired reads and singletons. In this case, we recovered 175 ‘un-mapped’ reads.

8- With this new set of ‘un-mapped’ reads, perform steps 1 and 2.

9- Post-mapping results and visualization of the ‘un-mapped’ reads dispersion along the reference sequence.


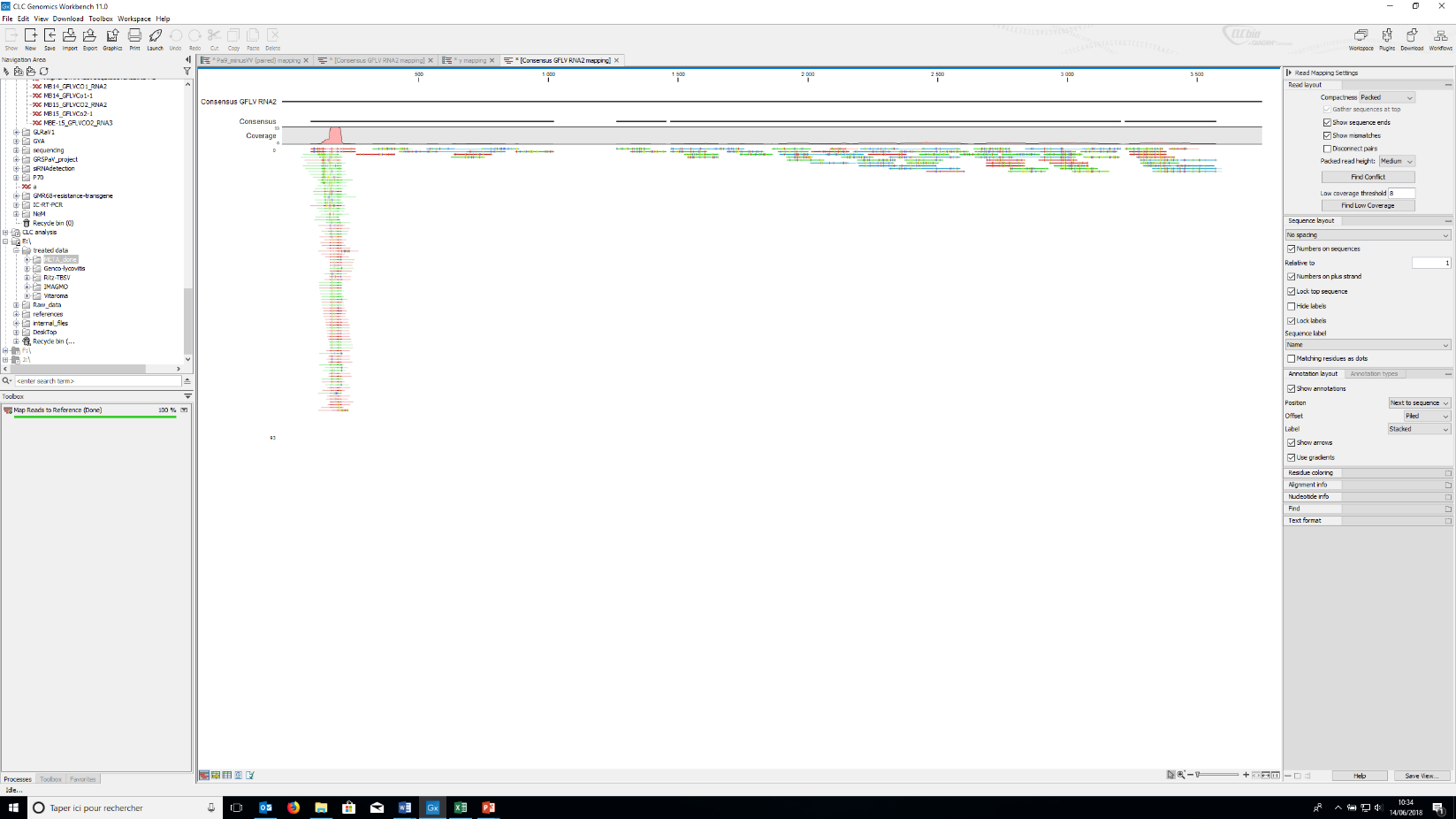

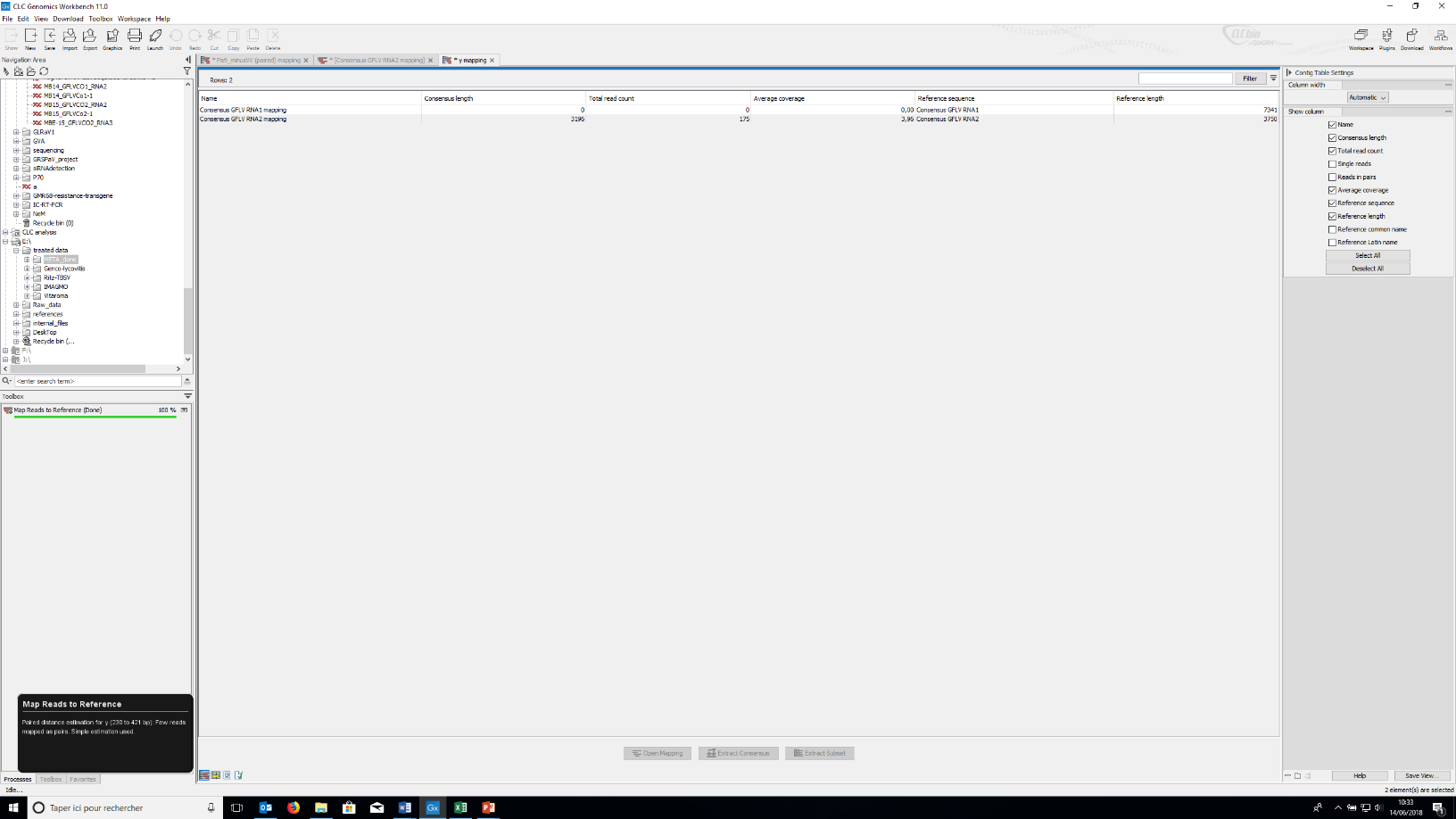


Here, all 175 previously recovered ‘un-mapped’ reads cover now 85% of the GFLV RNA2 consensus sequence, with a depth of around 10 (not taking into account the over-mapped region of the genome). Both numbers are higher than the 50% coverage and the depth close to 1 cut-off to be considered as a negative sample in step of the protocol presented in Figure 2.

3


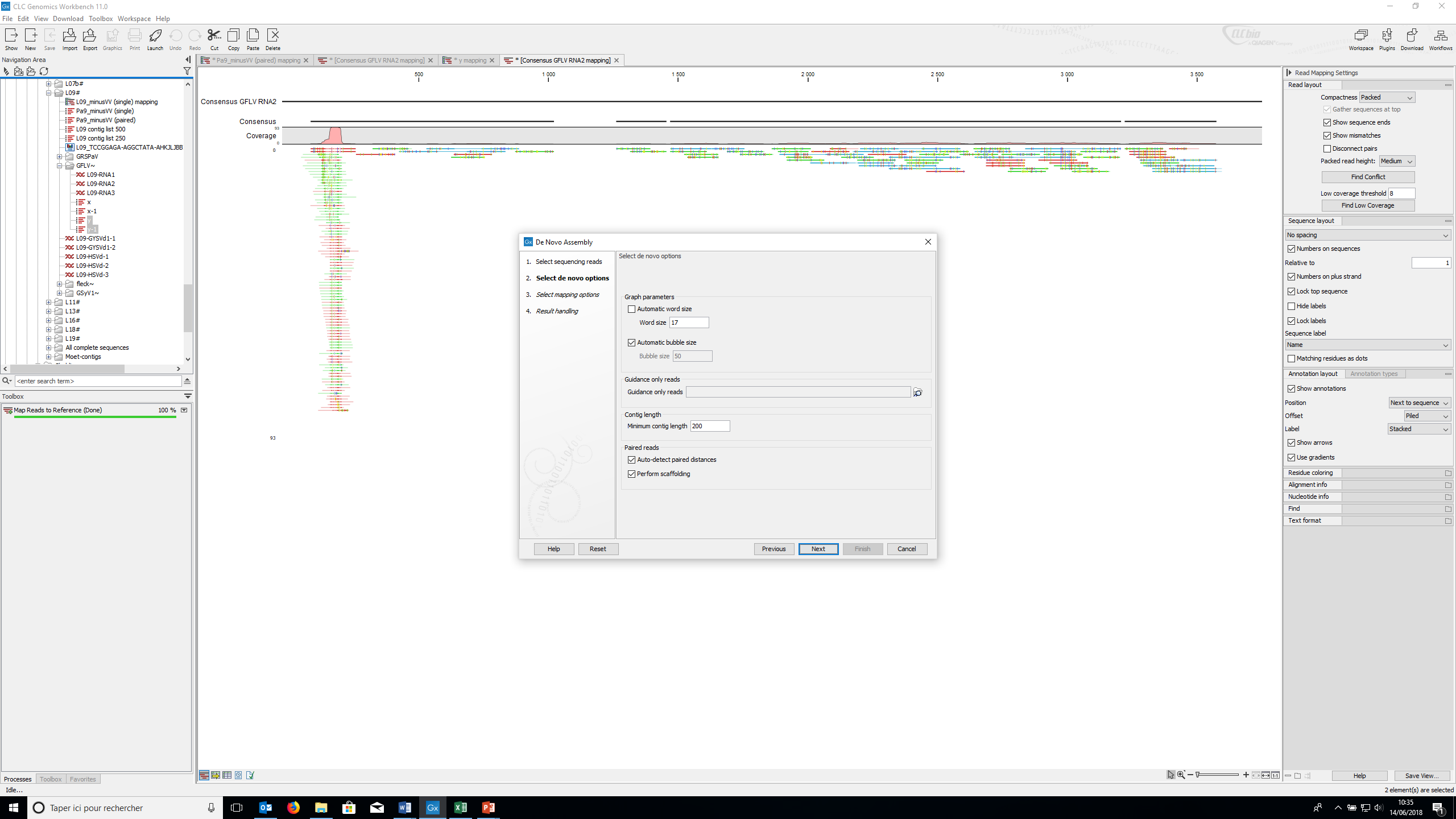
10- Perform a *de novo* assembly with loose parameters to take into account and assemble as many reads as possible.


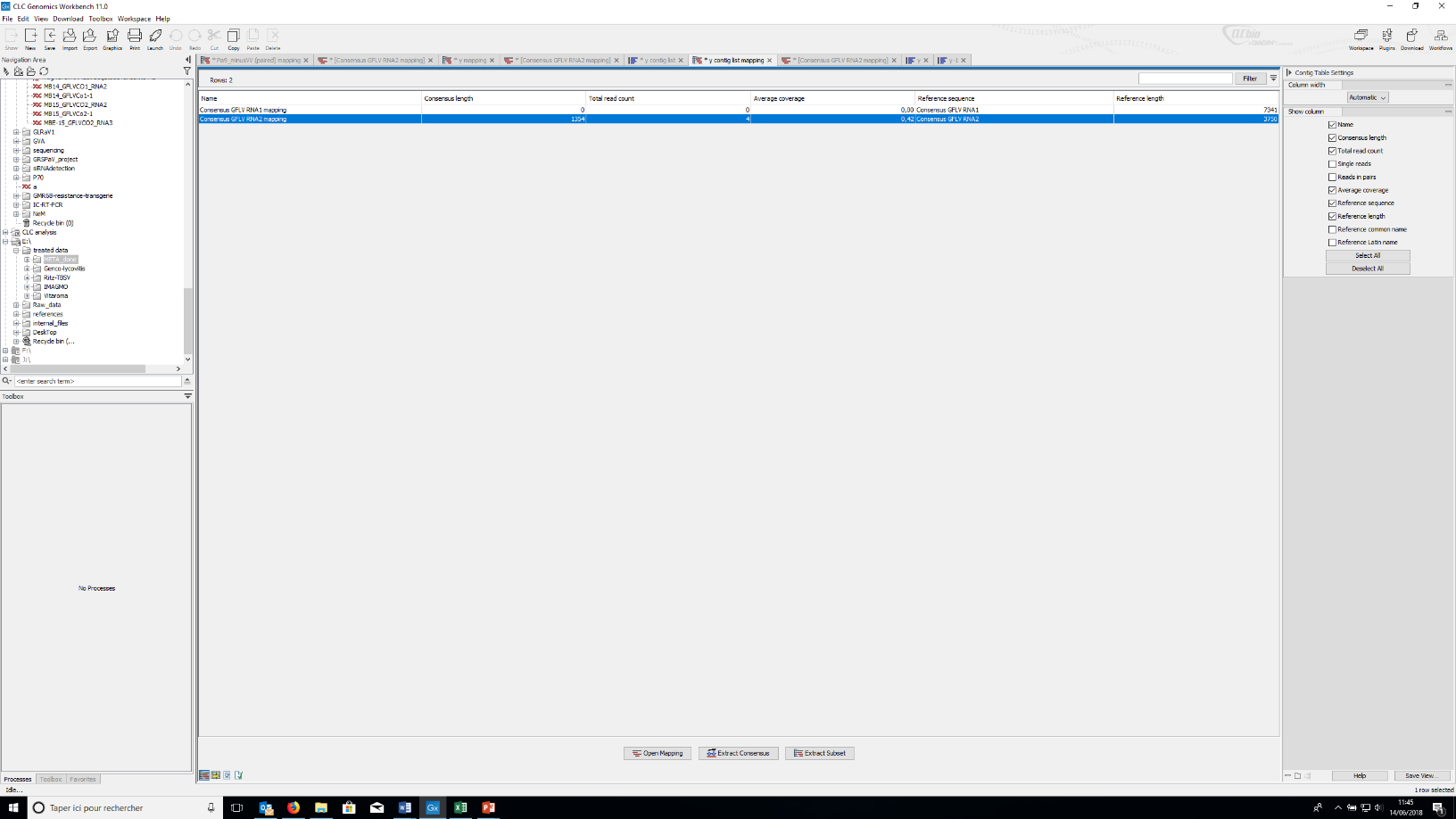
11- Finally map the contigs being assembled onto the same reference sequence with relaxed parameters as in steps 1 and 2.


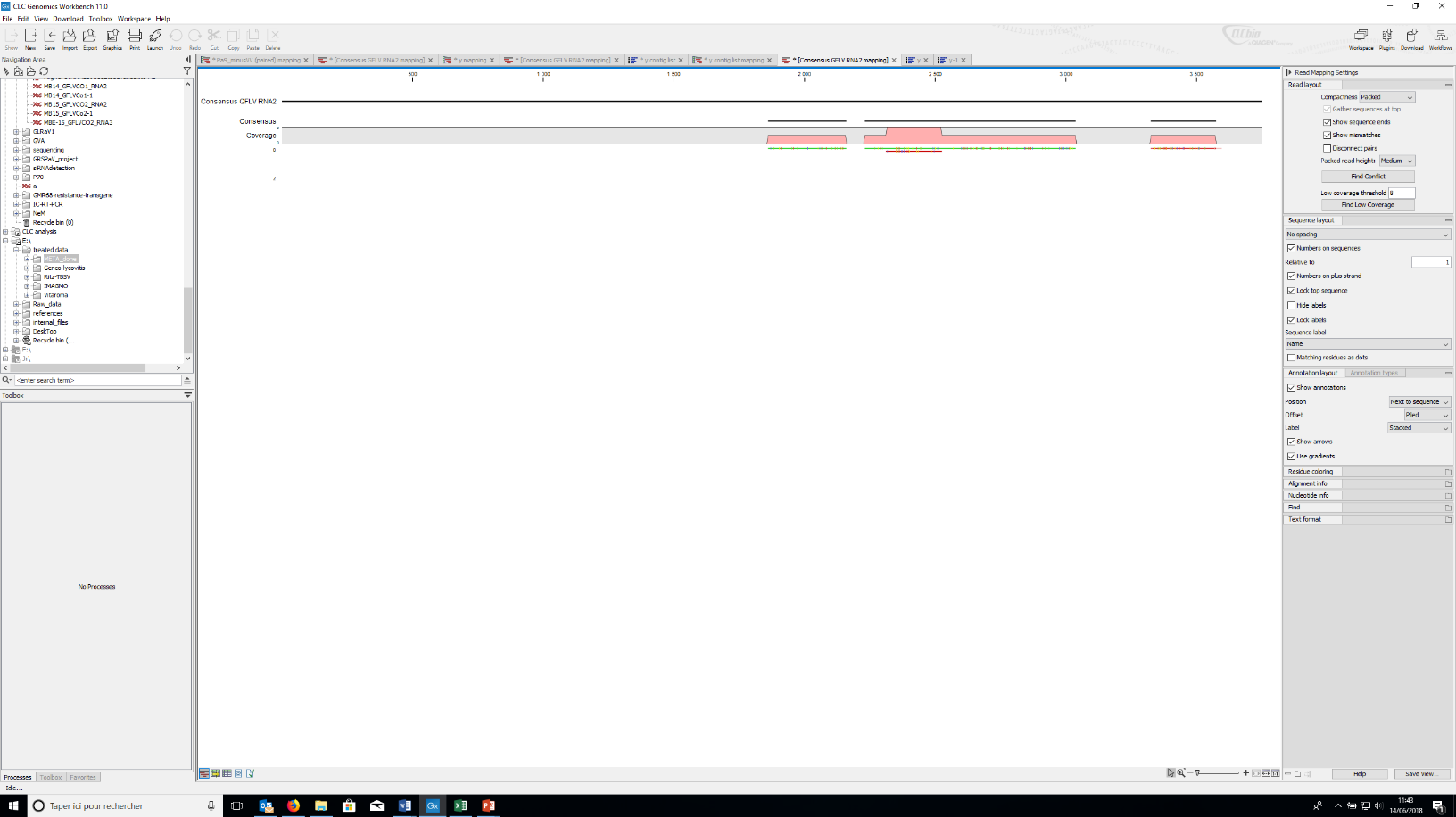


Here, four contigs were assembled which covered 36% of the reference sequence. This result defines sample Pa9 as a negative sample for the presence of GFLV RNA2 sequences.
