# Supplementary material for: Comparison of Serological and Molecular Methods With High-Throughput Sequencing for the Detection and Quantification of Grapevine Fanleaf Virus in Vineyard Samples
Source: Front Microbiol. 2018 Nov 22;9:2726. doi: 10.3389/fmicb.2018.02726 (PMC6262039; doi:10.3389/fmicb.2018.02726)
Supplement: Supplementary file 7 [file Data_Sheet_2.docx]

**Table S1: List of grapevine samples used for HTS in this study.** Lane 1 corresponds to RNAseq performed at the Get-PlaGe Genotoul plateform facility in July 2017, with twenty samples being multiplexed within a single lane. Lane 2 was multiplexed with the IMA-GMO project [Hily et al., 2018c] and sequencing was performed at the Get-PlaGe Genotoul plateform facility in September 2015. Lane 3 corresponds to smallRNAseq performed by Fasteris in July 2016. NT : Not tested.

**Table S2: List of GFLV isolates used in this study and GenBank accession number.**


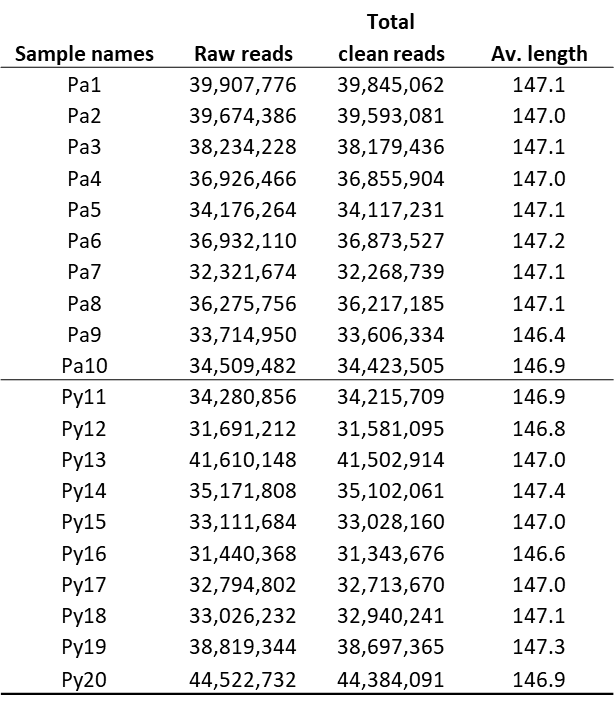


**Table S3: Raw reads**, total clean reads post quality check and trimming, and the average length for the RNAseq libraries obtained from 20 grapevine samples.


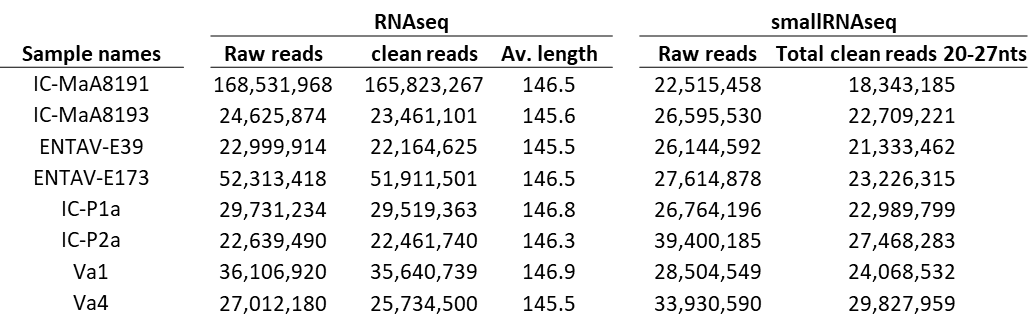


**Table S4:** Raw reads, total clean reads used in the RNAseq and smallRNAseq libraries for comparative study of eight grapevine samples.


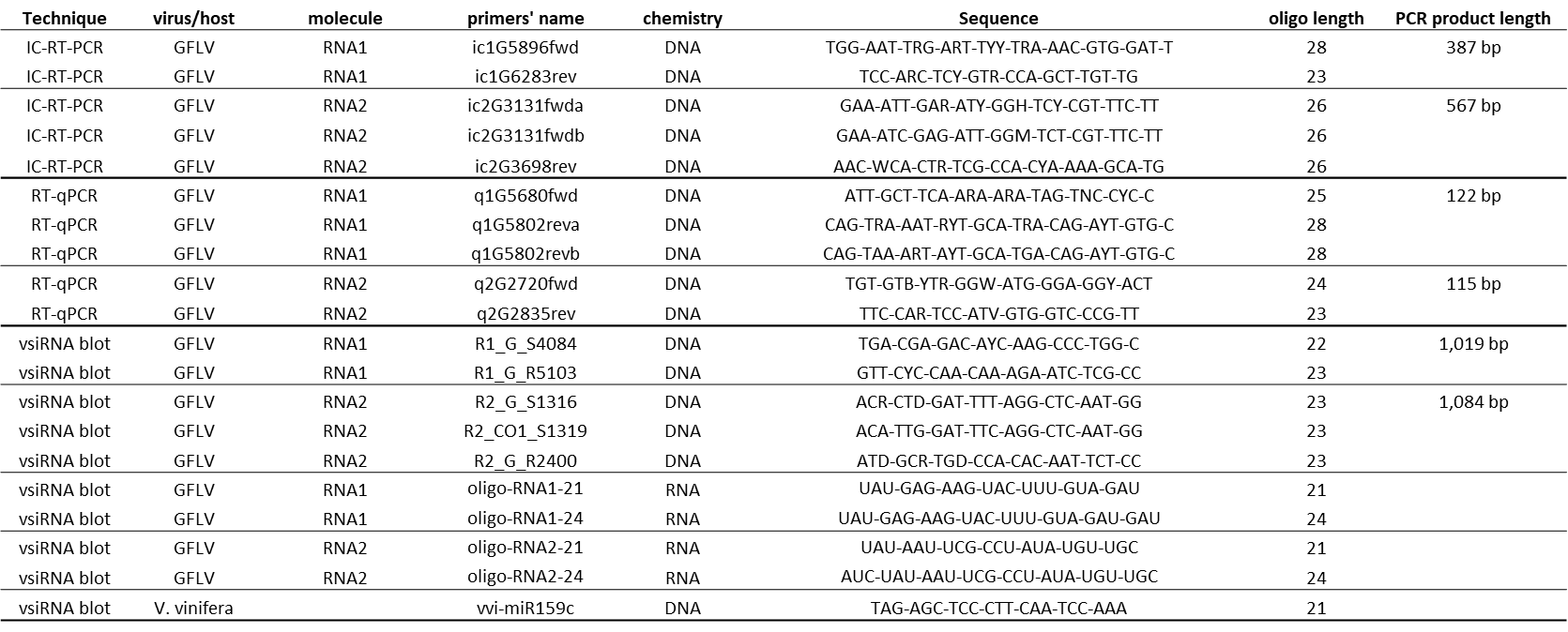


**Table S5:** List of primers used in this study either to detect GFLV by PCR-based methods or via the design of probes for blots.

**Table S6:** Number of reads mapping to GFLV RNA1, RNA2 and RNA3 consensus sequences. Grapevine samples positive (+) or negative (-) for GFLV following intra-lane contamination protocol are indicated.


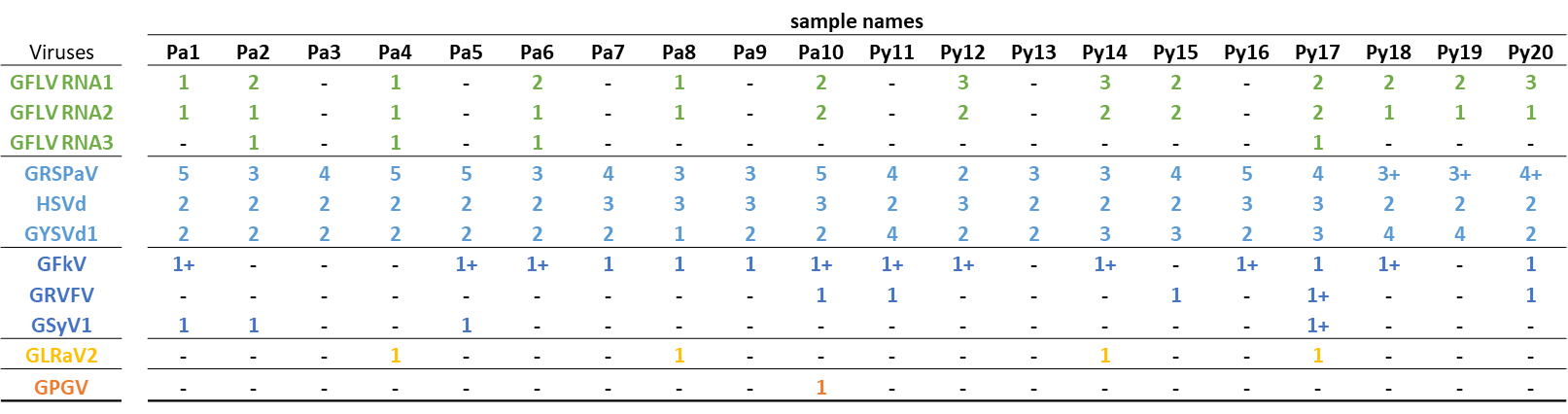


**Table S7**: Infection status of 20 grapevine samples from two vineyards in the Champagne region of France. Numbers correspond to complete to near complete sequences assembled in *de novo*. The presence of distinct but incompletely assembled variants are indicated (+). For GFkV, the genome was not fully assembled due to a high cytosine content.

**
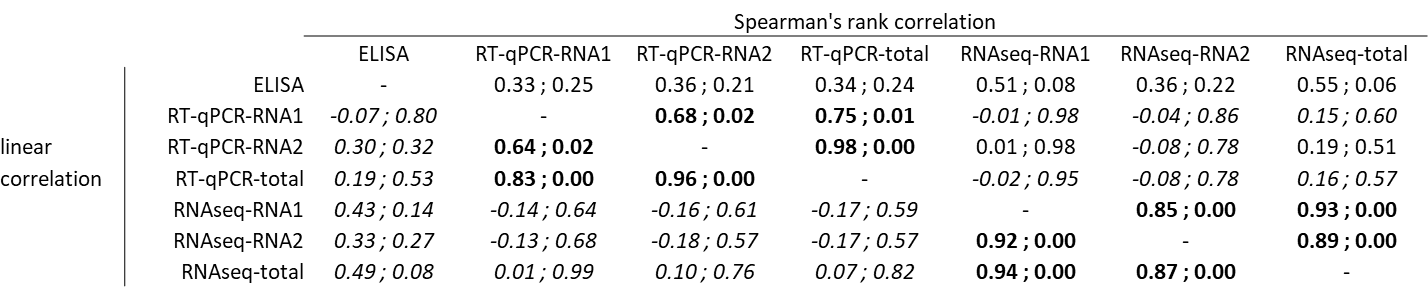
**

**Table S8: Pearson (linear) and Spearman’s rank correlations to compare quantification techniques.** Coefficient and P-value are shown (coefficient; P-value). If statistically supported (P-value<0,05), data are in bold. ‘Total’ corresponds to the sum of RNA1 + RNA2 data.

**Figure S1: Genetic diversity of the RNA1 and RNA2 sequences of the five GFLV isolates (**Table 4) chosen to represent the viral diversity. Analyses were performed by CLC Workbench software v11.0. After alignment, phylogenetic trees were created based on the Neighbor Joining (NJ) algorithm with bootstrapping analyses of 1,000 replicates. These sequences were selected to generate probes for vsiRNA blots.

**Figure S2: GFLV detection in twenty samples from two vineyards in Champagne**. (A) GFLV RNA1 and RNA2 molecules were detected separately by IC-RT-PCR and electrophoresis on 1.5% agarose gels, and (B) Both RNA molecules were observed together by vsiRNA blot analysis for which miR159 was used as a loading control.

**Figure S3: Depth and coverage of RNAseq and smallRNAseq datasets for some grapevine samples to illustrate their infection status, part I, II and III**

**Supplementary data sheet 1: GFLV intra-lane contamination detection from RNAseq libraries**
